# Microfluidic paper analytic device (μPAD) technology for food safety applications

**DOI:** 10.1063/5.0192295

**Published:** 2024-05-02

**Authors:** Soja Saghar Soman, Shafeek Abdul Samad, Priyamvada Venugopalan, Nityanand Kumawat, Sunil Kumar

**Affiliations:** 1 Division of Engineering, New York University Abu Dhabi, Abu Dhabi, P.O. Box 129188, UAE; 2 Department of Mechanical Engineering, New York University, Brooklyn, New York 11201, USA

## Abstract

Foodborne pathogens, food adulterants, allergens, and toxic chemicals in food can cause major health hazards to humans and animals. Stringent quality control measures at all stages of food processing are required to ensure food safety. There is, therefore, a global need for affordable, reliable, and rapid tests that can be conducted at different process steps and processing sites, spanning the range from the sourcing of food to the end-product acquired by the consumer. Current laboratory-based food quality control tests are well established, but many are not suitable for rapid on-site investigations and are costly. Microfluidic paper analytical devices (μPADs) are a fast-growing field in medical diagnostics that can fill these gaps. In this review, we describe the latest developments in the applications of microfluidic paper analytic device (μPAD) technology in the food safety sector. State-of-the-art μPAD designs and fabrication methods, microfluidic assay principles, and various types of μPAD devices with food-specific applications are discussed. We have identified the prominent research and development trends and future directions for maximizing the value of microfluidic technology in the food sector and have highlighted key areas for improvement. We conclude that the μPAD technology is promising in food safety applications by using novel materials and improved methods to enhance the sensitivity and specificity of the assays, with low cost.

## I. INTRODUCTION

The food we consume today is the product of local and global ecosystems where fresh and prepared food items undergo multiple processing, storage, and/or transportation steps. Keeping the food safe from pathogens, allergens, toxic chemicals, and adulterants is essential for maintaining a healthy population, and quality control at each step of food production and storage is critical. Unsafe food is a leading cause of malnutrition, especially in children, elderly, and the sick.^1^ As per a report by WHO, around 600 × 10^6^ incidents of illnesses and 420 000 deaths are recorded every year due to the consumption of unsafe food.^2^ In the United States alone, annual loss of USD 17.6 billion is attributed to medical costs, productivity losses, and death due to foodborne illnesses.^3^ Foodborne pathogens, toxins, allergens, and food contaminants contribute to illnesses, and in severe cases, even death. It is a challenge to detect and analyze these harmful components of food in a rapid, sensitive, and user-friendly way.

Food hygiene and safety from production to consumption can be ensured by management systems such as Hazard Analysis and Critical Control Point (HACCP), an international alliance.^4^ Here, food safety is addressed through the analysis and control of biological, chemical, and physical hazards from raw material production, procurement, and handling, to manufacturing, distribution, and consumption of the finished product. According to the Food and Agriculture Organization (FAO) of United Nations and the International Commission on Microbiological Specifications for Foods (ICMSF), the principles of HACCP are applicable to all phases of food production, including basic husbandry practices, food preparation and handling, food processing, food service, distribution systems, consumer food handling, and consumption. The most important concept underlying the HACCP system is that of the prevention of food hazards rather than inspection. The control of processes and safety conditions comprises the critical control point (CCP) elements with methodical, flexible, and systematic application of the appropriate science and technology for planning, controlling, and documenting food safety.^5^ HACCP protocols contain risk analysis, risk assessment, and risk management of agricultural products and food materials.^6^ In general, the food safety protocols follow the traditional and highly standardized laboratory methods for testing prescribed by FAO, US Food and Drug Administration (FDA), United States Department of Agriculture (USDA), and other national and regional agencies, but they also implement newer detection techniques as approved.^7^

First conceptualized in 2007, paper-based microfluidics continue to be an expanding research field, providing an innovative method for fluid handling and sample analysis for a variety of applications, including clinical diagnosis and environmental monitoring.^8,9^ In recent years, researchers have recognized the growing potential of microfluidic paper-based analytical devices (μPADs) and have developed promising devices for food analysis. They found that the μPADs allow easy, rapid, and cost-effective point-of-need screening of food materials. The fundamental working principle of microfluidic paper analytic device (μPAD) technology is to miniaturize the analytical device on a paper substrate and conduct the analysis using microunits of liquid reagents while maintaining the accuracy of detection. In food analysis, the testing reliability of μPADs could reach up to a correlation coefficient of R^2 ^= 0.99.^10^

μPADs are made up of different types of natural papers and modified papers. Paper is a ubiquitously available cellulose material that helps in economizing the μPAD production. μPADs possess several advantages over traditional microfluidics. They are compatible with commonly used biological and chemical reagents for food analysis. μPADs use capillary and gravitational forces for the absorption and flow of liquid samples without any complex additional active flow control devices, and the white background of paper helps in clear visualization of the results in colorimetric assays. Compared to other analytical device manufacturing, techniques for fabricating paper-based microfluidic devices are relatively simpler, such as plotting with an analog plotter, ink jet etching, plasma treatment, paper cutting, wax printing, ink jet printing, flexography printing, screen printing, 3D printing, and laser treatment.^8^ Based on the application, the fluid flow in a μPAD is controlled and guided by fabricating microfluidic channels or wells on the paper. The microfluidic channels or wells are made by creating patterns of hydrophilic and hydrophobic contrasted regions on the paper.^11^ The highly porous nature of paper offers high surface to volume ratio, which allows the absorption of more reagents and samples, aiding in better contact between reagent molecules while mixing. Compared to other food analytical devices and machines, μPADs require less foot space and they are easy to transport with less cold chain maintenance. Moreover, μPADs offer easy waste disposal options as most of them can be disposed off by incineration.

**FIG. 1.**
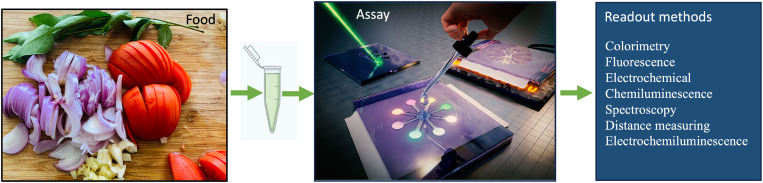
μPADs offer easy, affordable, reliable, and quick detection of food hazards such as pathogens, adulterants, allergens, and toxic chemicals. μPADs use methods such as colorimetry, fluorescence, electrochemical reactions, chemiluminescence, spectroscopy, distance measuring, and electrochemiluminescence for the detection and analysis.

This review focuses on the applications of μPADs in food safety. A brief account on the different μPADs fabrication techniques and designs, along with their advantages and limitations, is presented. Subsequently, the fluid flow control techniques and readout mechanisms, such as colorimetric, electrochemical, fluorescence, chemiluminescence (CL), and electrochemiluminescence, are described. This is depicted in Fig. 1. In the end, we have concluded with the future research areas and opportunities for highlighting the importance of μPAD-based microfluidic technology in the food safety sector.

## II. FOOD SAFETY

**FIG. 2.**
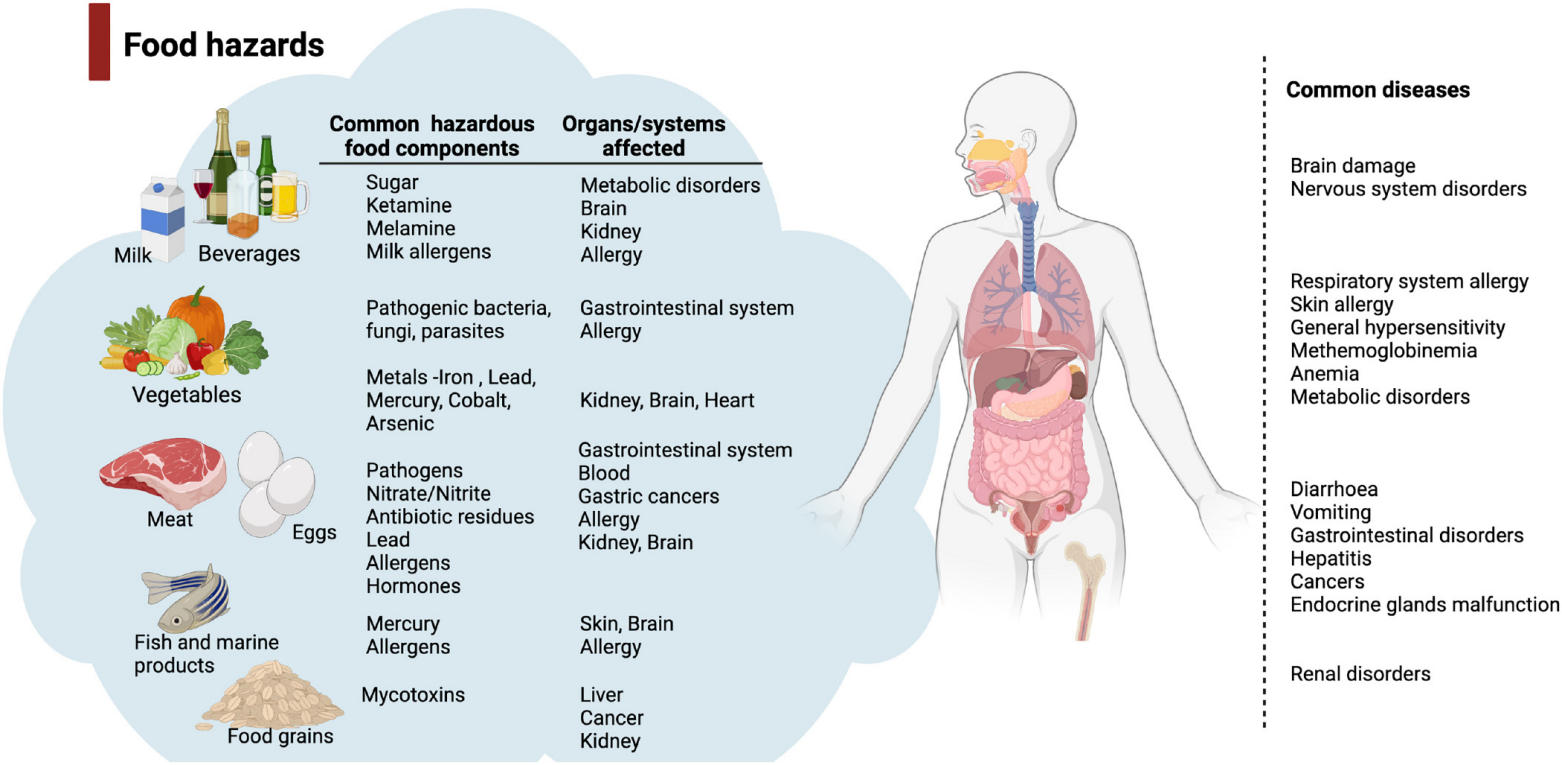
Foodborne pathogens, microbial toxins, food allergens, antibiotics and hormonal residues, food preservatives and additives, chemical toxicants, pesticides, and herbicides are the common food hazards. They cause acute and chronic organ toxicities and diseases affecting different body systems. Diarrhea, vomiting, and gastrointestinal disturbances are the most common acute symptoms observed in food poisoning, while different types of cancers are the chronic conditions caused by unsafe food. Illustrations created with BioRender.com.

Food safety remains one of the most important health concerns globally and is particularly challenging in resource limited areas. Considering the risks associated, timely detection of food hazards plays a critical role in controlling foodborne health threats. Quick, easy-to-use, and economical analytical tools are essential to detect harmful materials in food. The common food hazards adversely affecting the organ systems are foodborne pathogens and microbial toxins, food allergens, antibiotics and hormonal residues, food preservatives and additives, chemical toxicants, pesticides, and herbicides (Fig. 2).

### A. Foodborne pathogens

The major pathogens associated with food poisoning are bacteria, viruses, parasites, and fungi. A few representative pathogens causing severe foodborne illnesses are *Bacillus cereus*, *Staphylococcus aureus*, *Proteus vulgaris*, *Escherichia coli* O157:H7, *Coliforms*, *Campylobacter*, *Listeria monocytogenes*, *Salmonella* spp., *Cronobacter sakazakii*, *Vibrio* spp., *Aspergillus* spp., *Fusarium* spp., *Penicillium* spp., etc.^12,13^ The traditional culture techniques for the detection and identification of foodborne pathogens require 5–7 days to complete. Molecular detection techniques, such as polymerase chain reaction (PCR) and MALDI-TOF to identify the pathogens, are tedious and require well equipped laboratories and trained personnel. This demands a more effective method for pathogen detection, where μPADs become relevant.

**FIG. 3.**
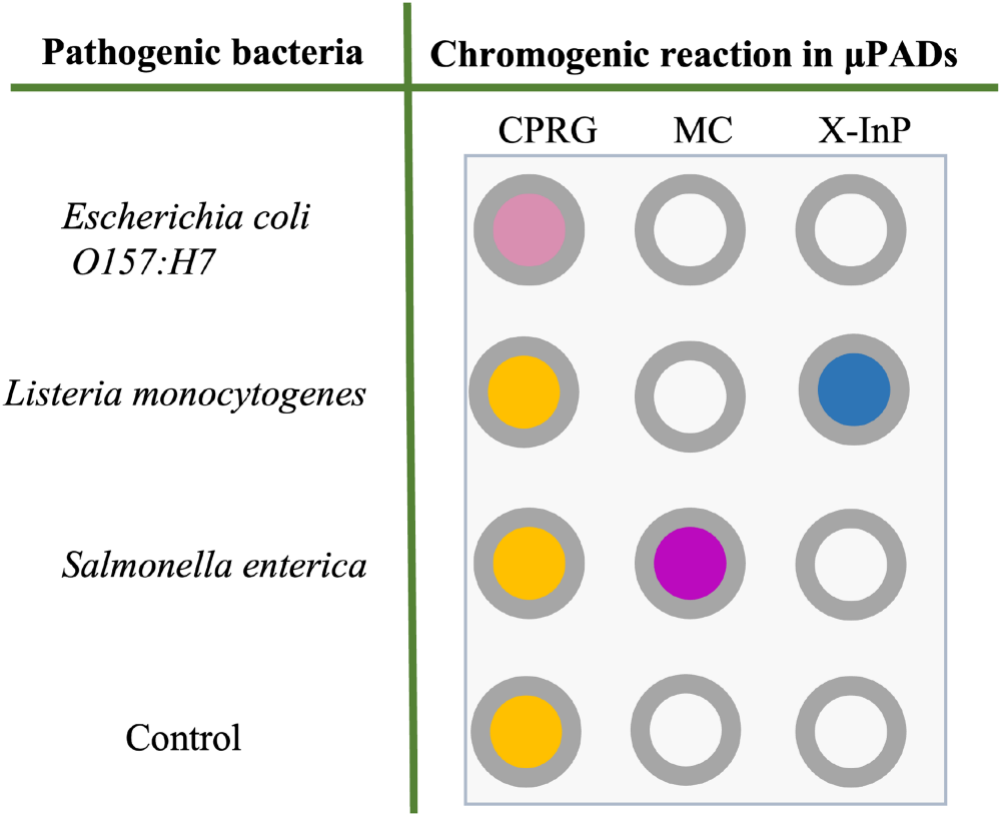
Detection of three potent foodborne pathogens using a chromogenic assay on μPADs. Bacterial specific enzymes and specific substrates were used for the detection. Cross-reactivity study tested the selectivity of each bacterial enzyme–substrate pair. The color change is specific to the enzyme and the substrate.

Similar to other testing methods, pathogen detection in μPADs includes sampling, treatment, assay, signal detection, and analysis. Most of the bacterial species produce enzymes that can be detected using a suitable substrate to develop specific color reaction in the paper matrix. Using this principle, the wax printing method was used on filter paper to develop a spot assay for the detection of *Escherichia coli O157:H7, Salmonella enterica,* and *Listeria monocytogenes* to analyze food samples.^14^ Three enzyme–substrate pairs were used for the detection of the above pathogens. *Escherichia coli* was detected using β-galactosidase with chlorophenol red β-galactopyranoside (CPRG), producing a red-violet color in positive samples.^15^ *Salmonella enterica* was detected by the reaction of bacterial esterase with 5-bromo-6-chloro-3-indolyl caprylate (MC) substrate giving a purple color. The enzymatic reaction of phosphatidylinositol-specific phospholipase C from *Listeria monocytogenes* with 5-bromo-4-chloro-3-indolyl-*myo*-inositol phosphate (X-InP) developed a blue color.^16,17^ This detection method reduced the bacterial enrichment time in media before conducting the assay to 12 h or less. The assay could detect as low as 10^1^ colony-forming units/cm^2^ bacteria in meat samples^18^ (Fig. 3).

Artificially synthesized single-stranded nucleic acid (DNA or RNA) probes are known as Aptasensors. Aptasensors are created using a method called the Systematic Evolution of Ligands by Exponential Enrichment (SELEX). SELEX employs a library of random oligonucleotide sequences and involve multiple rounds of amplification in an exponential way, with the goal of isolating at least one aptamer with high affinity to the target pathogen.^19,20^ Aptasensors directed to a specific bacteria can interact with the surface of the bacterial cells directly to enable the detection without complex sample preparation procedures. Aptasensors incorporated in μPADs could detect two food poisoning pathogens; *Escherichia coli O157:H7* and *Salmonella typhimurium* simultaneously.^21^ Polystyrene (PS) microparticles assembled with gold nanoparticles (Au NPs) were used for fabricating μPADs. A salt-based nanoparticle aggregation mechanism could produce stable colorimetric signals after the sample addition and drying. Images were captured using an iPhone camera and the greyscale images were analyzed using a python algorithm. The colorimetric results showed linearity over a wide concentration range of cultures of *Escherichia coli O157:H7* and *Salmonella typhimurium* (10^2^–10^8^ CFU/ml). This technique shows the ability of multiplexing and rapid testing on μPADs while maintaining high assay sensitivity.

A gold-based sensing platform integrated with magnetic nanobead–peptide probes in a paper strip was developed to detect the presence of *Staphylococcus aureus* in food samples.^22^ The reaction detects the cleavage orchestrated by the *Staphylococcus aureus* specific proteases and dissociation of the magnetic nanobeads from the sensor surface. The gold sensing platform allows the detection of bacteria in food products such as ground beef, turkey sausage, lettuce, and milk spiked with pure bacterial broth culture. The test resulted in a limit of detection of 7–100 CFU ml^–1^ of bacteria. This assay is simple to perform, label-free, economical, quick to carry out within minutes, and suitable for other types of bacteria and stable for 6 months when kept cold.^23,24^

**FIG. 4.**
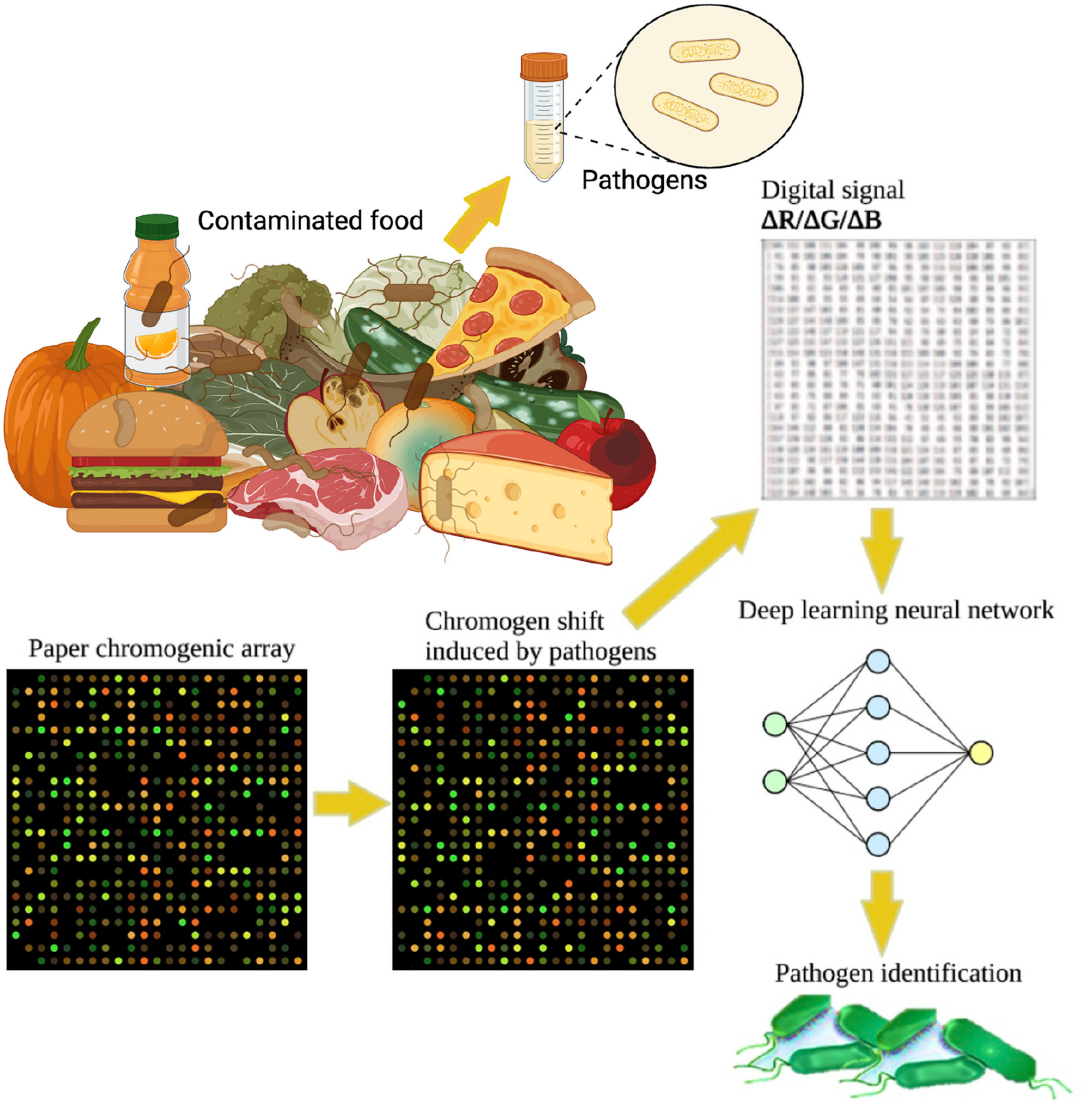
A μPAD paper chromogenic array (PCA) to spot individual pathogens in the presence of nonpathogenic background microflora in food materials was developed based on the production of volatile organic compounds (VOCs) by individual pathogen. The color pattern shift was analyzed digitally. The pattern was used to construct a deep feedforward neural network, which showed 93% accuracy to detect individual bacteria. Illustrations created with BioRender.com.

Food materials contain a mixture of pathogenic and nonpathogenic bacteria. A new version of μPAD-based chromogenic array, named paper chromogenic array (PCA) to spot individual pathogens (*Listeria monocytogenes*, *Salmonella enteritidis*, and *Escherichia coli* O157:H7) or multiple pathogens in the presence of nonpathogenic background microflora on food was developed.^25^ This array integrates a machine learning approach for pathogen detection based on the emitted volatile organic compounds (VOCs) by individual pathogen.^26,27^ Grade 1 cellulose chromatography paper PCA was fabricated into which 22 chromogenic dye spots were infused along with standard color dots (Fig. 4). When the array is exposed to volatile organic compounds emitted by the pathogens of interest, the dye spots exhibited remarkable color changes and pattern shifts. The pattern was analyzed digitally and used to construct an advanced deep feedforward neural network. After training, the network demonstrated excellent performance in identifying pathogens with 93% accuracy.

Coliforms are notorious pathogens responsible for water contamination. If consumed, they can cause extreme illness to fatality. An open-channel μPAD was created by direct printing of omniphilic channels on an omniphobic, fluorinated paper, capable of lysing and detecting coliforms. The lysing step helps in the disruption of bacterial cell wall to release its content to mix with the detection reagents. They used lysing cell mixture with high and low surface tension liquids. This μPAD device demonstrated the flow and control of both high and low surface tension liquids such as cell lysing agents. The μPAD device could detect *Escherichia coli*, at a concentration as low as ∼10^4^ CFU ml^−1^, using the bacteria specific, β-galactosidase enzyme.^28^

### B. Microbial toxins

The presence of microbial toxins in food materials is a serious problem worldwide. Toxins produced by bacteria, fungi, algae, and marine organisms can be extensively present in food materials processed and stored in unsafe conditions. Foodborne toxins include mycotoxins, marine biotoxins, cyanogenic glycosides, and toxins occurring in poisonous mushrooms. Aflatoxins and ochratoxins are the most important mycotoxins that are present in food. Aflatoxins are produced by molds, especially by Aspergillus flavus and Aspergillus parasiticus.^29^ There are four aflatoxins—B1, B2, G1, and G2—as well as two metabolites of aflatoxins M1 and M2.^30^ Aflatoxin B1 is the most common and potent of all the aflatoxins and is a common milk contaminant and carcinogen, which can cause liver diseases, mutations, and cancer.^31,32^ The standard method to identify mycotoxins is by analyzing via labor intense methods like high-performance liquid chromatography (HPLC) and high-performance liquid chromatography-tandem mass spectrometry (HPLC–MS/MS) or through regular ELISA kits.^33,34^ However, most of these methods could not be used directly for complex food matrixes and demands to develop robust techniques to detect food toxins.^29,35^

An automated μPAD-based competitive enzyme-linked immunosorbent assay (ELISA) to detect aflatoxin B1, with a detection limit of 60 femtograms or 0.1 ng/ml was developed. The μPAD used a dissolvable sucrose valve for fluid flow control and used only submicroliters of samples (0.6 *μ*l).^36^ The test achieved high sensitivity and minimized sample volume simultaneously by using a new sample-loading method: directly applying the sample solution at the zones on the device that had been prepared with an antibody-conjugated enzyme before immersion in a running buffer. The method provides high sensitivity, small sample volume, equipment-free measurements, low-cost operation, and user-friendliness. This approach could be adopted to analyze other small-sized toxin molecules in different types of food materials.^37^

A colorimetric assay on a microfluidic paper device was developed for the rapid detection of aflatoxin B1, using specific aptasensors. Aptameric–gold nanoparticle conjugate was physically adsorbed on a μPAD and sample containing aflatoxin B1 was allowed to flow over the μPAD. The nanoconjugate was characterized using UV-vis spectroscopy, and dynamic light scattering for measuring hydrodynamic diameter and zeta potential. The assay could detect 1 *μ*M to 1 pM of aflatoxin B1 with a limit of detection of 10 nM in standard samples.^38^

In another study, a paper-based microfluidics chip to measure the mycotoxin, deoxynivalenol was devised using a colorimetric competitive immunoassay, using gold nanoparticles, with a detection range of 0.01–20 ppm.^39^ A portable paper-based microfluidic aptasensor is established to visually detect mycotoxins, zearalenone, and ochratoxin A, simultaneously. The hydrophobic paper matrix is made using laser printing and heat treatment. In this device, the analytes at the sample zone can migrate into separate detection zones through dual-channels. The recognition of toxins is possible by the specific aptamers that destroy fluorescence resonance energy transfer (FRET) from dual-color upconversion nanoparticles (UCNPs) to Cu-TCPP nanosheets and result in green and blue fluorescence recovery. Zearalenone and ochratoxin A could be measured in the sample by capturing fluorescent images and analyzing the corresponding RGB value via a smartphone, with limits of detection down to 0.44 and 0.098 ng/ml, respectively.^40^

### C. Food allergens

Food allergy is an important health concern affecting upto 10% of the population. Food allergy can cause mild to severe symptoms, and in extreme cases, food allergy can lead to anaphylaxis, which is a life-threatening allergic condition. Currently, there is no cure for food allergy. Management of food allergy includes allergen avoidance, quick detection, and emergency treatment. Rapid and accurate detection of allergens in food is crucial for effective curbing of grave outcomes. The most common food allergens are eggs, milk, peanuts, tree nuts, soy, wheat, crustacean shellfish, and fish. Most of the available allergen tests are time consuming, costly, and available only in specialized facilities. μPADs serve as a promising technology to address these challenges.

A three color multiplex lateral flow immunoassay (xLFIA) to detect common allergenic milk casein, ovalbumin, and hazelnut proteins was devised on μPADs. The antibodies against the allergens were individually adsorbed onto gold and silver nanoparticles to produce specific-colored probes on nitrocellulose membrane (Hi-flow plus 180) strips. The strips were inserted in a LFIA device comprising of three lines, each line representing for one allergen. The xLFIA could identify allergens in commercial biscuits as low as 0.1 mg/l.^41^ This microfluidic paper-based immunoassay for allergen detection shows good fluid control and have multiplexing capabilities.

A lateral flow immunoassay (LFI) μPAD for quick detection of allergic protein in food samples was fabricated using a modified cellulose material. The device could detect the allergen in 15 min, including the sample preparation time. The sample flow rate was optimized by adjusting the geometrical patterns on μPAD. This μPAD could detect as low as 1 ppm ovalbumin—a major egg allergen, in different food samples.^42^ A sandwich immunoassay strip test with nitrocellulose membrane was developed to detect Bowman–Birk inhibitor, a type of antinutritional factor present in soybean, causing indigestion and stunted growth in human and animals.^43^ The test could detect allergen concentrations up to 0.5 *μ*g/ml visually, and 0.23 *μ*g/ml with a TSR3000 Membrane Strip Reader (BioDot, USA).

### D. Food additives and preservatives

Food additives and preservatives are mostly chemical substances, widely used for enhancing the color, flavor, texture, and shelf life of food. These chemicals are non-toxic when used in limited quantities, but may lead to toxicity and ill health if consumed in large quantities. In this section, we describe the μPADs developed for testing food additives and preservatives.

**FIG. 5.**
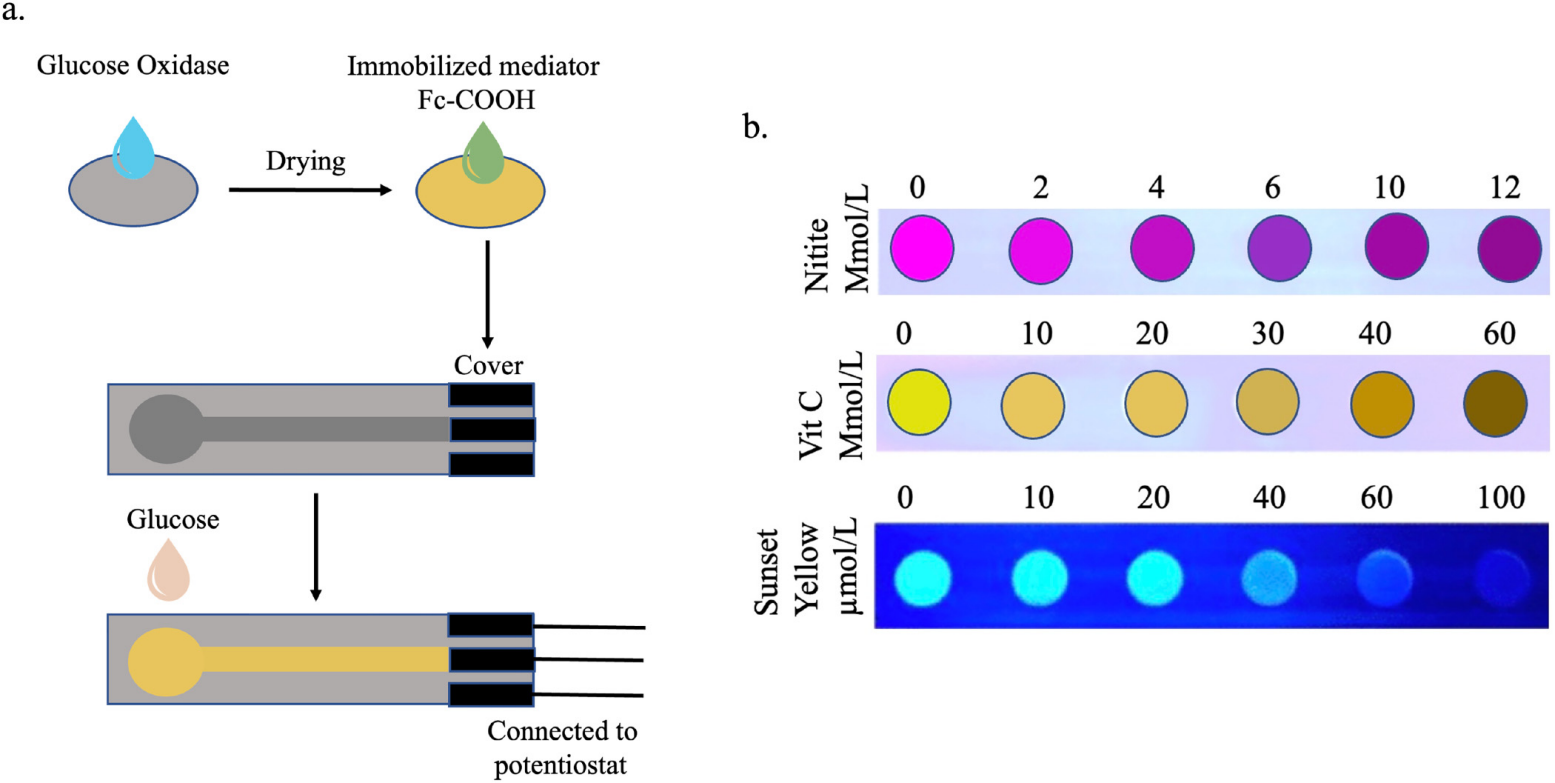
(a) Amperometric biosensing was used for the detection of glucose from commercial beverages. An amperometric biosensor works based on the oxidation and reduction of an electroactive species on a biosensor surface using immobilized enzymes such as glucose oxidase. (b) A paper-based chip was used for visual determination of food additives at different concentrations. Fluorescent carbon dots (CDs), namely, red-CDs, blue-CDs, and yellow-CDs were visualized under UV spectrum to detect nitrite, vitamin C, and sunset yellow in a sample.

Sugary beverages are the single largest source of calories and added sugar in the U.S. diet.^44,45^ In developing countries, sugary drink consumption is rising exponentially due to widespread urbanization and beverage marketing. Added sugar in foods serves as a sweetener, preservative, texture modifier, fermentation substrate, flavoring and coloring agent, and bulking agent. Close monitoring of the sugar content is required in commercial drinks, as these sugar-loaded beverages can increase the risk of type 2 diabetes, heart disease, and, thus, premature death.^46^ low-cost, sustainable biosensors using cellulose paper substrate have been developed to measure glucose in commercial beverage samples. The device was made up of hydrophilic cellulose paper disk impregnated with immobilized glucose oxidase enzyme placed on top of a screen-printed carbon electrode. Amperometric biosensing was used for the detection here. An amperometric biosensor functions based on the oxidation and reduction of an electroactive species on a biosensor surface using immobilized enzymes. Amperometry measures the electric current vs time (*i*–*t*) when a constant electric potential is maintained. The Glucose biosensor could accurately analyze very low sample volumes (5 *μ*l) of commercial beverages with a limit of detection comparable to high-performance liquid chromatography.^47^ The reaction chemistry is illustrated in Fig. 5.

Inkjet printing is a simple and commonly used method to fabricate μPADs. Researchers have developed inkjet printed paper sensors to analyze food additives, such as food coloring agents like sunset yellow, food preservatives, e.g., nitrite and vitamins, e.g., vitamin C. The device used fluorescent carbon dots (CDs) for the detection. CDs are nanomaterials, which can emit or quench florescence when binding with specific molecules.^48,49^ In this method, an optimized ink is prepared using organic solvents (e.g., absolute ethanol), polyethylene glycol to slow the drying, and surfactants like FS3100 and SE-F to control the viscosity. The optimized ink was filled into a clean printer cartridge (Hewlett Packard or HP) connected to a computer, and a pre-designed pattern was printed using an HP Deskjet 2628 printer on the No. 2 medium-speed flow qualitative filter paper. After patterning, the microfluidic chips were dried in the temperature range of 60–100 °C. The method demonstrates the fluorescence-based qualitative detection of food additives at various concentrations using a UV lamp at 365 nm [Fig. 5(b)]. The performance of the inkjet printing is highly reliant on the optimal formulation of the printing inks.

Benzoic acid is a frequently used preservative in pickled food and beverages. A Whatman qualitative filter paper-based microfluidic chip device was fabricated for the detection of benzoic acid in food using the Janovsky reaction theory. Janovsky reaction was performed in the following manner in the reaction zones of the μPAD. The circular reaction zones of the μPADs were implanted with 5 N sodium hydroxide and dried at 30 °C for 20 min. The benzoic acid sample was converted to 3,5-dinitrobenzoic acid using KNO_3_ and H_2_SO_4_ reagents at 40 °C for 40 min and was added on the reaction zones. Then, the μPAD device was transferred to a portable detection system and heated at a temperature of 45 °C for 20 min on a hot plate to carry out the chemical reaction. The resulting color change from light to dark brownish-orange shade in the detection zone is imaged using a Complementary Metal Oxide Semiconductor (CMOS) camera. The color change was analyzed using a RGB analysis software after transferring to a smartphone. The color change was proportional to the benzoic acid concentration in the sample.^10,50^ Researchers have analyzed 21 different types of commercial food samples, including sauces, processed fruits, and dried and pickled vegetables using this method. The standard deviation of the results was not more than 6.6% compared to the HPLC method and the coefficient of correlation across the tests was equal to R^2^ = 0.9953.

**FIG. 6.**
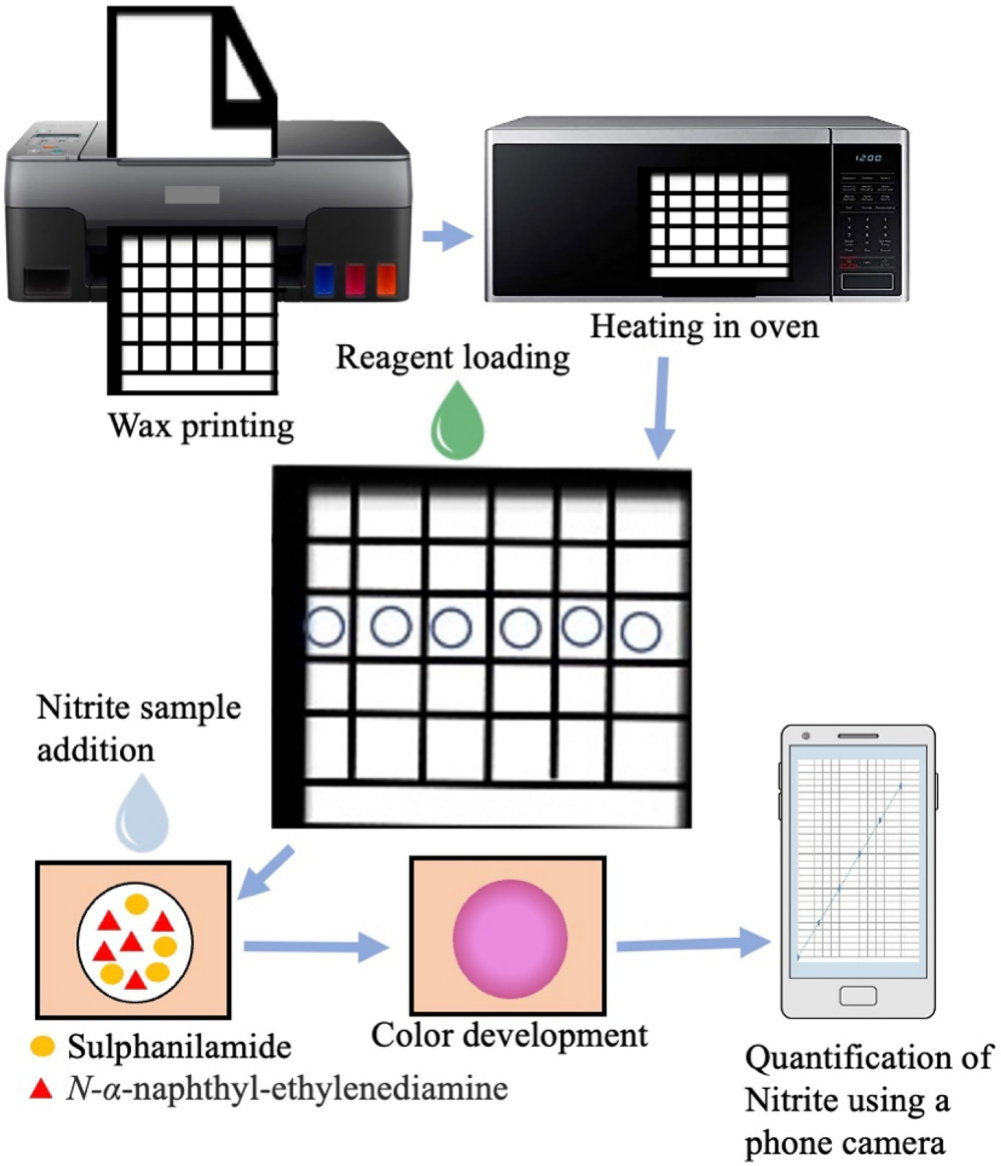
Nitrite is a food preservative. A schematic of colorimetric determination of nitrite. The detection was performed through Griess reaction carried out on μPADs. The intensity of color developed was scanned and analyzed using a mobile device.

Nitrite is a meat preservative used to extend the shelf life and to maintain the fresh appearance. High nitrate content in meat and vegetables can cause methemoglobinemia and gastric cancers. The colorimetric determination of nitrite is possible through the Griess reaction carried out on μPADs.^51,52^ In this reaction, nitrite reacts with sulfanilamide and produces positively charged diazonium salt. This salt couples with *N-α*-naphthyl-ethylenediamine to produce a magenta azo dye compound. The formation of azo compound is directly proportional to the nitrite concentration in the sample. The image of the color developed was captured using a phone camera and analyzed using ImageJ. The test can be carried out quickly within 15 min, with a sensitivity of 1.1 mg kg^−1^ (Fig. 6). The microfluidic reaction on μPADs could detect nitrite in meat samples including processed meat, pork, ham, sausages. and drinking water.^53,54^

### E. Chemical toxicants and adulterants

The most common method of food adulteration in solid food is the addition of substances, such as sand, ground stones, and pebbles to food grains and used tea leaves addition to tea. Metallic compounds, such as mercury, lead, arsenic, cobalt, and cadmium, are also found as common food adulterants. Moreover, deliberate contamination of pure forms of food products with low quality foreign substances, such as rancid oils, and altered species of meat are emerging concerns. In the last decade, numerous μPADs have been developed to detect food adulterants such as chemical toxicants in food safety sector. We will discuss some of them in this section.

Melamine is a nitrogen containing substance illegally added to milk, infant formula, and pet food to artificially increase the protein value of the food.^55–57^ The addition of 1% melamine in food causes an artificial elevation of food protein content to 4%.^58^ Melamine in excess amount can form insoluble melamine-cyanurate crystals in the kidney and cause renal damage.^59–61^ Colorimetric detection of melamine adulteration in milk was detected on a Whatman filter paper-based visual sensor using Triton X-100 modified, stabilized gold nanoparticles (AuNP). The chemical reaction happens between the Melamine and the AuNPs through the ligand exchange with citrate ions on the surface of AuNPs, leading to the Triton X-100 being removed. The AuNPs aggregate and produce a color change from a wine red to blue depends upon the concentration of the Melamine. This color change causes a shift in absorption peak while measuring using UV-vis absorption spectroscopy. The method could accurately detect melamine as low as 5.1 nM in milk samples, which is a lower value of Melamine concentration as per food safety regulations.^62^ The naked eye could detect 1.0 μM Melamine in milk samples. The same technique was modified to suit the rapid testing in field conditions using smartphone-based detection of color change.

Researchers developed μPAD devices to detect Mercury poisoning in salmon fish. For the detection, the μPADs were coated with modified gold nanoparticles (AuNPs). The AuNPs have been modified with *N*,*N*′-bis (2-dihydroxyethyl) dithiooxamide (HEDTO) and coated on a hydrophobic-hydrophilic barrier created by Triethoxymethylsilane. Upon adding mercury containing samples, HEDTO-AuNPs formed aggregates and showed a color change from red to blue on μPADs. The detection was facilitated by capturing the images of color change using a smartphone camera. The images were analyzed using Adobe Photoshop CS6 image processing software. The technique could offer a limit of detection of 15 nM of mercury from food samples.^63^

μPADs were fabricated and tested for 10%–50% (v/v) palm oil adulteration in sunflower oil. The devices were prepared in the form of circular discs and rectangular channel strips using simple cutting and crafting technique for visible colorimetric detection. The images were captured using UV-vis spectrophotometry and the average grayscale intensity data were analyzed. The coefficient of determination of the tests was 0.9464, indicating a good empirical fit and suitability of the method for quantitative detection. The assay device was evaluated for stability of signal over a time span of six days after the test. The images were captured every day and analyzed for grayscale intensity values using ImageJ software. The signal remained fairly constant over the test duration, allowing the time flexibility of data analysis after the test.^64^

One modification in the μPADs technology is the development of electrochemical microfluidic paper-based analytical devices integrated with nanotechnology, called EμPADs. EμPADs report the detection and quantification of anesthetic drugs, such as ketamine in alcoholic and non-alcoholic drinks, using electrochemical sensing. Ketamine is a criminally abused drug in party beverages, and in incidences of robbery to sedate the victims. In this method, the EμPAD surface was coated with zeolite-nanoflakes and graphene oxide nanocrystals (Zeo-GO). When the test beverage (alcoholic drink or fruit juice) was applied to the circular working zone of the EμPADs, the nanocrystals of ZeO-GO EμPAD showed electro-oxidation of ketamine presented in the beverages, thus causing a change in the signal intensity while sensing. The change in the intensity was correlated to the drug concentration in the beverages. This method showed a swift response time of 2 s, with a limit of detection of 0.001 nM/ml and a wide range of detection from 0.001 to 5 nM/ml.^65^ Similar types of μPADs were devised for the detection of misused drugs such as Estazolam and Clenbuterol.^66,67^

**FIG. 7.**
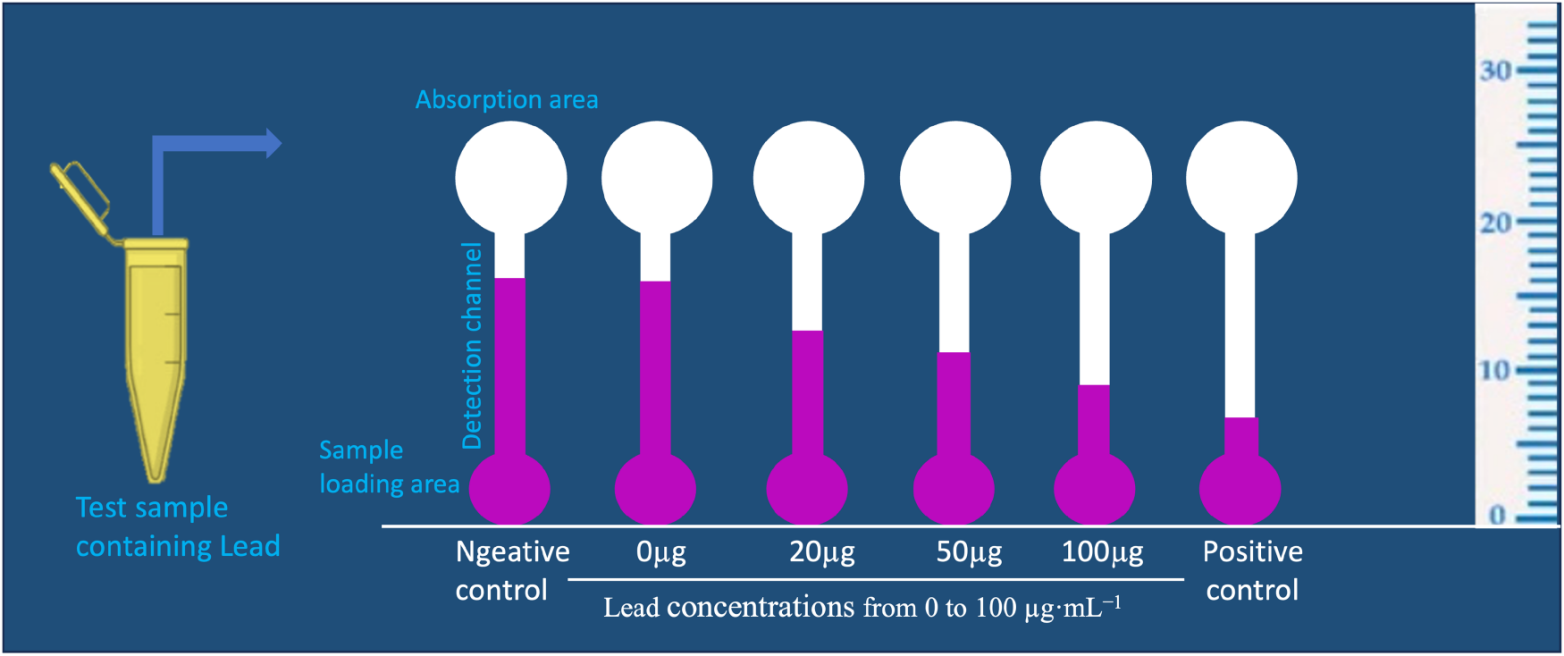
Here, it shows a prototype of distance paper-based analytical device (dPAD) for analyzing lead (Pb) in food samples. The decrease in the color distance on the immobilized dPAD observed when introducing Pb at concentrations ranging from 0 to 100 μg ml^−1^ with 0.8 mmol l^−1^ carminic acid in 0.1 M hydroxyethyl piperazine ethane sulfonic acid (HEPES) buffer. The reactions were incubated at ambient temperature for 5 min before analysis.

World Health Organization (WHO) classifies lead (Pb) as a toxic metal of greatest public health concern.^68^ Pb exposure accounts for more than 1 × 10^6^ deaths each year and causes the loss of 24.4 × 10^6^ disability-adjusted-life-years. Pb causes irreversible acute and chronic neurotoxicity and immune dysregulations.^69,70^ Pb toxicity occurs through food crops, preserved eggs, and drinking water. A wax printed distance measuring paper-based analytical device (dPAD) has been developed for the detection of Pb in preserved century eggs. The assay uses the competitive binding chemistry between Carminic Acid (CA) and polyethyleneimine (PEI) to detect Pb in food samples. The assay principle is based on the binding property of CA to Pb under appropriate pH conditions. The CA–Pb complex would be trapped in the sample-loading area, allowing only free CA to wick through the hydrophilic detection channel toward the absorption area (Fig. 7). The device was patterned with a sample reservoir and a hydrophilic channel containing a colorimetric indicator reactive to the sample. When the sample is added and flows through the channel, the indicator generates a colored band, whose length can be measured by keeping a ruler along the length of the channel. The length of the colored bands was inversely proportional to the concentration of the analyte added. The method allows us to read and interpret the results without using any sophisticated readout system. dPAD showed good linear correlation of *R*^2^ value 0.974 for measuring Pb, within the ranges of 5–100 *μ*g ml^−1^. The results from the dPAD were comparable to the measurement obtained by atomic absorption spectroscopy.^71^ dPAD has the potential to be applied in food processing in developing countries, and it does not require laborious instruments and procedures.

### F. Pesticides and herbicides

The use of organophosphorus (OP) pesticides, such as carbamates, is common in agriculture and forms a major chemical hazard in food. Carbamate is listed as an endocrine disruptor compound, capable of causing hormonal abnormalities if consumed.^72^ Analyzing these compounds in food materials is important in the food processing step. The use of μPADs technology has been developed for the colorimetric determination of carbamate pesticides based on the inhibition of acetylcholinesterase (AChE) enzyme by the pesticide while reacting with a specific chemical substrate.^73–75^ The reaction chemistry is as follows: acetylthiocholine iodide substrate is hydrolyzed into thiocholine and acetic acid by AChE. The thiocholine base reacts with 5,5′-dithiobis-(2-nitrobenzoic acid) (DTNB) to generate a yellow color detectable at 405 nm. The intensity of the yellow color developed is inversely proportional to the pesticide concentration. The images were captured using a desktop scanner and analyzed for color intensity using ImageJ software. The device was designed as a spot-array assay with a well diameter of 10 mm detection zone on the patterned paper. The device was distinguished with color codes on hydrophobic and hydrophilic zones, blue color was identified as the hydrophobic zone, and yellow for the reaction developing zone. The design was printed on the sheet of Whatman chromatography Paper using a wax printer. The hydrophobic barrier was created on the paper by heating the paper at 150 °C for 2 min in an oven to melt the wax to impregnate the wax throughout the thickness of the paper in the defined design. The AChE inhibition was determined by plotting a calibration curve of the pesticide concentration against percentage inhibition.^76,77^ The results were compared with no pesticide controls and different concentrations of pesticides. Another important point to be noted in assay using enzyme kinetics is that high concentrations of pesticides may inhibit the complete activity of AChE, shown as the absence of yellow color. Therefore, a standard detection range is to be followed while conducting these assays.

A sensitive surface-enhanced Raman scattering (SERS) method using gold nanoparticles (Au NPs) is developed to detect methyl parathion on fruit surface. The filter paper substrate was immersed in the prepared Au NP solution. The spectroscopic probe molecule 4-mercaptobenzoic acid (4-MBA) was used to evaluate the performance of the paper substrate for an optimized signal using a portable Raman spectrometer coupled with 785 nm laser. Then, the substrate was applied to detect methyl parathion standard solutions with a linear range between 0.018 and 0.354 *μ*g/cm^2^ with a limit of detection of 0.011 *μ*g/cm^2^. After standardization, the actual fruit peel sample spiked with methyl parathion was used to verify the test. The test recovery rate was 94.09%–98.72%, indicating high reliability in testing fruit samples without rigorous pretreatment. The method showed excellent reproducibility and stability.^73^

A colorimetric determination method for analyzing the widely used herbicide, glyphosate in food grains was developed on μPADs. Glyphosate is linked to disease conditions such as organ toxicity and cancer.^78^ The glyphosate detecting system showed improved selectivity and sensitivity by using Mn–ZnS quantum dot (QD) embedded molecularly imprinted polymers (MIPs) on μPADs. The detection of glyphosate is based on the oxidation of 2,2′-azino-bis(3-ethylbenzothiazoline)-6-sulfonic acid (ABTS) by H_2_O_2_ in the presence of Mn–ZnS QD-MIP. Glyphosate non-binding-Mn–ZnS QD-MIP generates ^•^OH from H_2_O_2_, producing a dark green color of the test zone. The binding of glyphosate to the Mn–ZnS QD-MIP turns off the generation of ^•^OH, resulting in light green color.^79^

### G. Hormonal and antibiotic residues in food

17β-estradiol is a potent anabolic steroid hormone derivative, illegally used to promote the growth of animals. The residues of 17β-estradiol in foods and milk can cause endocrine disruption through the food chain accumulation.^80,81^ Molecularly imprinted polymer (MIP) grafted paper-based devices were fabricated for the detection of 17β-estradiol in milk, with high reliability.^82^ The MIP's optimum synthetic conditions were optimized with the following reagent mixture: acetonitrile as the solvent, 17β-estradiol as the template molecule, (3-aminopropyl) triethoxysilane (APTES) as the functional monomer and tetraethyl orthosilicat (TEOS as the cross linker in the ratio of 1:12:12. This assay showed a limit of detection of 0.25 μg l^−1^ for spiked milk samples. These paper-based devices and methods can develop into a reliable new platform for high-throughput, sensitive, specific, and multiplex assay in food monitoring.

Antimicrobial resistance is a global threat due to the nonjudicious use of antibiotics to treat bacterial infections in food producing animals. Predictive statistical models estimated 4.95 × 10^6^ deaths associated with bacterial antimicrobial resistance in 2019.^24^ A number of cost-effective μPAD-based diagnostic devices have been developed to detect antibiotics residues in milk, egg, and meat.^67,83,84^ The presence of oxytetracycline and norfloxacin residues in pork was determined using metal complexation on microfluidic paper-based analytical devices (μPADs).^84^

The μPAD technology showed breakthrough platforms to revolutionize food quality analysis with the advantage of equipment-free detection and analysis, at low cost. In Secs. III–V, we will look into the μPAD fabrication techniques, flow control methods, and detection techniques.

## III. FABRICATION OF μPADs

The process of fabrication of paper-based microanalytical devices includes the selection and preparation of hydrophilic porous medium and the formation of isolated hydrophilic micro-zones. This could be achieved either by patterning hydrophobic boundaries to enclose hydrophilic zones or by cutting and physically separating the required zones from the rest of the porous medium. The different fabrication techniques can be broadly classified into indirect or direct patterning of hydrophobic barriers, or physical isolation of hydrophilic zones.

### A. Indirect patterning methods

In this type of patterning, the designs are first made on an intermediate masking substrate and are subsequently transferred to the porous paper. Fabrication techniques where a pre-built mask is required to selectively shadow the paper substrate are photolithography, screen printing, chemical vapor deposition (CVD), plasma treatment, wax coating, and spraying.

#### 1. Lithography

In Whiteside's revolutionary innovation of modern paper-based analytical devices for micro-fluidic applications, the devices were fabricated using conventional photolithography process.^85^ Chromatographic paper coated with SU-8 2010 grade photoresist was exposed to UV light. A quartz photomask with the patterns was placed over the paper for the lithography step. Barriers and channels with sub-micrometer resolution were reported using this method. The photolithography procedure involves multiple process steps, use of organic solvents, and needs expensive, pre-prepared masks, and cleanroom environment. Further, the wet chemical treatment and multiple baking steps result in corrosion of the porous medium and compromise its natural wettability.^86^ To relax the sophistications associated with conventional photolithography process, a Fast Laser Activation of Sheets (FLASH) method, employing custom-formulated inexpensive photoresist, instantaneously printed transparent sheet masks, and sustainable low-cost UV sources was developed.^87^ Further, several lithography-assisted, hybrid techniques have been invented to avoid the wet chemical processing of paper sheets. Yu and Shi developed μPADs by embossing a lithographically created parafilm master on paper sheets.^86^ In another approach, He *et al.* used OTS-coupled silanized paper and exposed it to DUV (254 nm) lithography in an O_3_ ambience. The silane bonds in the unmasked regions were, thus, selectively decomposed by the UV radiation.^88^ The instrumentation required for the process makes the lithography-based approaches not feasible for the rapid and mass production of μPADs within a budget and resource limited setup.

#### 2. Screen printing

**FIG. 8.**
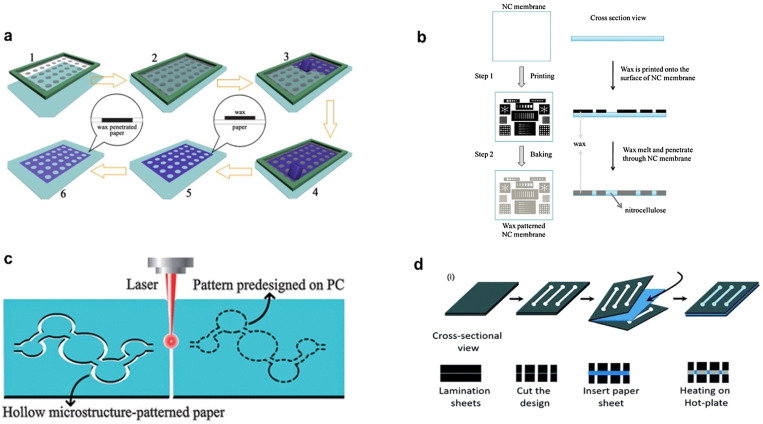
Schematic of different techniques used in fabrication of μPADs. (a) Screen printing using a custom-built stencil as a mask. Reproduced with permission from Wang *et al.*, Biosens. Bioelectron. **31**(1), 212–218 (2012).^90^ Copyright 2012 Elsevier. (b) Wax printing process for direct patterning of hydrophobic barriers. Reproduced with permission from Yao *et al.*, Anal. Chem. **82**(1), 329–335 (2010).^91^ Copyright 2010 American Chemical Society. (c) Laser cutting, as a direct patterning method for barrier creation. Reproduced with permission from Nie *et al.*, Analyst **138**(2), 671–676 (2013).^92^ Copyright 2013 Royal Society of Chemistry. (d) Cut and heat plastic lamination process, as a hybrid method for the hydrophobic barrier creation. Reproduced with permission from Kumawat *et al.*, Lab Chip **22**(18), 3377–3389 (2022).^11^ Copyright 2022 Author(s), licensed under a Creative Commons Attribution (CC BY) license.

Screen printing is a simple, rapid, and inexpensive method used for the fabrication of μPADs, first introduced by Dungchai *et al.*, as wax screen printing.^89^ Figure 8(a) shows the steps involved in the process of screen printing of μPADs. In this method, first, a stencil screen with the desired patterns is prepared.^90^ Holding the stencil tightly over the paper substrate, paraffine wax is rubbed over the screen, to transfer patterns to the paper. The excess wax is then squeegeed off. In a later heating step, the wax is melted and impregnated into the paper matrix to create hydrophobic barriers. Typically, a mesh of nylon on an aluminum or wood frame is used as the screen. Apart from wax, a variety of hydrophobic materials such as polystyrene, poly(methyl methacrylate) (PMMA), polydimethylsiloxane (PDMS), polycaprolactone, polylactic acid, varnish, and rubber-latex derivatives have been experimented as the ink for screen printing.^93–97^ Although screen printing is a simple, effortless, and universal method, there are certain downsides, such as poor resolution of the printed patterns, lack of automation, and requirement of multiple screens.^98^

#### 3. Etching/deposition/plasma treatment

Selective wet etching of silanized paper is a technique that involves low-sophistication, being free of expensive equipment and reagents. To achieve the chemical etching, Cai *et al.* used a tetramethyl orthosilicate (TMOS)-treated paper and aligned a cheap paper mask penetrated with NaOH solution (30% glycerol). The regions of the hydrophobic silanized paper, in contact with the masked regions with etching reagents, turned hydrophilic.^99,100^ In contrast, dry patterning techniques involve the surface modification of paper substrate by vapor phase deposition of hydrophobic materials or plasma assisted treatment. Chemical vapor deposition (CVD) is an additive process, which is useful for the solvent-free deposition of functional polymers, pure polymers, and inorganic compounds on paper substrate.^101–104^ The chemical precursors in gaseous form react at the surface of the paper and form a solid thin hydrophobic film. The masking for the deposition is attained either via patterns created through lithography or via physical blocking with metal or vinyl sheet. Selective plasma treatment is a dry, chemical-free method to alter the hydrophilic nature of the paper structure. μPADs are fabricated by selectively exposing the paper to a plasma discharge through metal masks.^105,106^ The plasma generation is accomplished by vacuum plasma reactors or portable corona generators. With the combination of plasma assisted fluorocarbon deposition and O_2_ plasma assisted etching, fully enclosed μPADs have been achieved.^107^ Both CVD and plasma-based methods allow solventless, substrate-independent way of the creation of hydrophobic barriers. However, the fabrication process involved in these techniques is relatively complex and requires costly reagents, equipment, and multiple masks.^108^ Other indirect patterning methods include dipping, lacquer spraying, embossing, or stamping of hydrophobic materials.^86,109–115^

### B. Direct patterning methods

In this type of hydrophobic barrier creation, designs defined using CAD software are directly printed on the paper surface using commercially available printers. The requirement for multiple masks, expensive reagents, and sophisticated equipment are thereby generally excluded.

#### 1. Wax printing

Wax printing is arguably the most extensively used technique by researchers for the fabrication of μPADs for food safety applications, including the detection of pathogens, neurotoxic residues, biotoxins, additives, and adulterants in food. In wax printing, the designs are directly printed on the paper surface using a wax printer, as shown in Fig. 8(b). With a subsequent heating step, the wax is melted and impregnated into the porous medium to form hydrophobic barriers.^91,116,117^ The overall process is rapid, minimal effort, and solvent-free. The limited resolution, the requirement of expensive wax, and the extra heating step are a few limitations of wax printing.^118^ Apart from these, the discontinuation of commercial wax printers from the market is the main challenge for the continued device fabrication using the technique.

#### 2. Inkjet printing

In the inkjet patterning method, the commercial inkjet printer is filled with a solvent ink, and the inkjet is used to define the boundaries enclosing hydrophilic zones.^119,120^ Alkyl ketene dimer (ADK), UV-curable ink, and hydrophobic solgel are some of the commonly used inks.^121^ The inkjet printing can be accomplished in two modes: (i) to impregnate hydrophobic ink barriers in the hydrophilic paper or (ii) to selectively etch and induce hydrophilic zones in a hydrophobized paper. Apart from the barrier creation, the inkjet method can be used to simultaneously print biomolecules, biomarkers, and immunoassay reagents on the paper microzones, thereby creating complete biosensing platforms.^122^ Hossain *et al.* first demonstrated the development of bioinks and bioactive papers for rapid detection of neurotoxins and pesticides in food.^123,124^ Later, omniphobic flouroalkylated (R^F^) papers printed with high resolution conductive patterns were developed by Lessing *et al.*^125^ Inkjet printing has become a powerful technique for defining immuno-chemical and conductive circuits directly on paper. The scalable, foldable, low-cost, and disposable printed circuits fabricated using inkjet printing extend their applications from healthcare to diagnostics and to high-performance electronics. One of the major drawbacks of this method is the slow process due to its inherent dot-by-dot printing approach, which is not suitable for rapid, mass production. Further, the involvement of hazardous solvents, the difficulty in instrumentation due to limited mechanical properties of the bio-inks, and multiple printing steps are some of the factors that need improvement.^121^

#### 3. Flexographic printing

Flexography is a direct roll-to-roll printing technique used for μPAD fabrication. An anilox roller is used to transfer a thin, uniform layer of hydrophobic ink to a flexible printing plate roller, which has the required designs in the form of relief patterns. Then, the ink from the raised portions of the printing plate is transferred to the paper substrate, fastened to an impression roller. Polystyrene or PDMS, dissolved in organic solvents, is used as hydrophobic ink for flexographic or roll-to-roll printing of μPADs.^126–128^ The hydrophobic property is decided by the number of layers printed and the penetration depth of ink is limited by the viscosity of solvent, vapor pressure, and solute content. The requirement of complex reagents and multiple roller plates are the drawbacks of this method.

#### 4. Laser printing

In the laser printing method, solid toner ink from a laser printer is used to create hydrophobic patterns on paper. The two-step fabrication process involves the automated printing of the design on paper and a post-baking step.^129,130^ The high temperature heating step and longer baking time required for the melting and impregnation of the toner ink is a drawback associated with this method.

#### 5. Laser direct-writing

Laser-based direct patterning is another fabrication technique, which employs photopolymerization of hydrophobized paper. Here, the paper is pre-treated with a photopolymer and then the laser beam is used to direct-write patterns on the paper, without any mask. The radiation-exposed regions undergo hardening by photopolymerization, thus forming the hydrophobic barrier, and the unexposed polymers are washed away using a solvent.^131^

### C. Physical isolation methods

Rather than the usage of hydrophobic materials, this approach relies on the physical isolation of the required hydrophilic zone from the rest of the porous medium by cutting boundaries using sharp tools or laser cutter. Paper sheets cut using a knife or scissors is the simplest way of creating a dipstick or chromatographic device. Laser-based cutting of paper sheets offers the speed, precision, scalability, and automation required for mass production capabilities [Fig. 8(c)]. CO_2_ laser is often employed for the controlled ablation of the cellulose structure and cut through the paper matrix.^92,132,133^ The physical isolation techniques create stand-alone hydrophilic patterns, which often require a supporting platform to provide mechanical strength. Kumawat *et al.* developed a method in which CO_2_ laser cutter is used for cutting features in plastic lamination sheets.^11^ A filter paper sheet is then placed between the lamination sheets and heated on the hot plate. The melting of the EVA from lamination sheets and its impregnation to the porous medium results in the barrier formation as outlined in Fig. 8(d). The fabricated devices have high mechanical strength against bending, folding, and tearing and are highly robust against strong acids, bases, and solvents. The technique presents a simple and low-cost fabrication process using widely available consumables and commercial tools for automated mass fabrication of μPADs. Table I shows the summary of some of the recent research based on the applications of μPADs for food safety.

**TABLE I.** Table showing the different fabrication techniques used for developing μPADs for food safety applications.

Year	Fabrication method	Detection scheme	Application in food safety
2009	Inkjet printing	Colorimetric assays	*Neurotoxic pesticides—bendiocarb, carbaryl, malathion* ^124^
	Inkjet printing	Colorimetric assays	*Neurotoxic pesticides—paraoxon, aflatoxin B1* ^123^
2011	Inkjet printing	Colorimetric assays	*Heavy metal ions—mercury, silver, copper, cadmium, lead, chromium, and nickel* ^134^
2012	Wax printing	Colorimetric assays	*Foodborne pathogens—Escherichia coli*, *Salmonella* spp., and *Listeria monocytogenes*^14^
	Wax printing	Electrochemical	*Quality control—food additives—ascorbic acid and sunset yellow in beverages* ^135^
2013	DUV lithography	Colorimetric assays	*Quality control—food additives—nitrite in food* ^136^
2014	Stamping	Colorimetric assays	*Quality control—food additives—nitrite* ^137^
	Inkjet printing	Colorimetric assays	*Quality control—food additives—nitrite and nitrate in water* ^54^
	Screen printing	Colorimetric assays	*Foodborne pathogens—Escherichia coli* ^94^
2015	Hybrid—wax/inkjet printing	Colorimetric assays	*Neurotoxic pesticides—paraoxon, malathion* ^138^
	Wax printing	Colorimetric assays	*Quality control—iodate in salt* ^139^
	Plasma treatment	Colorimetric assays	*Quality control—amylose in rice* ^140^
	Wax printing	Colorimetric assays	*Foodborne pathogens—Salmonella* ^141^
	Cutting/masking with adhesive tapes	Chemiluminescence	*Pesticide residue—DDV in vegetables* ^142^
	Stamping	Colorimetric assays	*Quality control—preservative additives—nitrite in ham, sausage, water* ^51^
	Wax printing	Fluorescence	*Heavy metal ions/antibiotic—silver/mercury, neomycin in water* ^143^
	Wax dipping	Colorimetric assays	*Heavy metals—copper, nickel, chromium in water* ^144^
	Screen printing	Colorimetric assays	*Heavy metal ions—copper in water, food, and blood* ^145^
2016	Screen printing	Colorimetric assays	*Neurotoxic pesticides—MPO and CPO in cabbage and green mussel* ^146^
	Wax printing	Chemiluminescence	*Heavy metal ions—chromium in natural water samples* ^147^
2017	Wax printing	Fluorescence	*Foodborne pathogens—E. coli* ^148^
	Wax printing	Colorimetric assays	*Adulteration—caramel in whiskey* ^149^
	Screen printing	Colorimetric assays	*Chemical residue—bisphenol A in food packets* ^150^
	Wax printing	Fluorescence	*Biotoxin—phycocyanin in water* ^151^
	Inkjet printing	Colorimetric assays	*Neurotoxic pesticides—paraoxon, trichlorfon* ^152^
	Wax printing	Electrochemical	*Adulteration—ketamine in beverages* ^153^
2018	Wax printing	Colorimetric assays	*Quality control—benzoic acid in food* ^10^
	Wax printing	Angle based readout and colorimetry	*Neurotoxic pesticides—DMMP* ^154^
	Screen printing	SERS	*Pesticide residue—thiram, thiabendazole, methyl parathion in fruits and vegetables* ^155^
	Hybrid—cutting /NP aggregation	Colorimetric assays	*Adulteration—melamine in milk* ^156^
	Photolithography	Chemiluminescence	*Mycotoxin—DON, ZEN, T-2, and HT-2 in cereals* ^157^
	Wax printing	Colorimetric assays	*Drug residue—clenbuterol in milk* ^158^
	Hybrid—wax/inkjet printing	Colorimetric assays	*Quality control/adulteration—protein, urea, and nitrite in milk* ^159^
	Photolithography	Fluorescence	*Food allergens and toxins—egg white lysozyme, ß-conglutin lupin, okadaic acid, brevetoxin* ^160^
	Hybrid—cutting/lamination	Colorimetric assays	*Adulteration—metamizole, paracetamol and midazolam maleate in whiskey* ^161^
2019	Wax printing	Chemiluminescence	*Quality control—antioxidants in food—gallic acid, quercetin, catechin, kaempferol, caffeic acid* ^162^
	Inkjet printing	Colorimetric assays	*Heavy metals—calcium in water* ^163^
	Wax printing	Hybrid/colorimetric assays	*Mycotoxin—DON in food/feed* ^39^
	Wax printing	Colorimetric assays	*Adulteration—neutralizers, urea, and detergents in milk* ^164^
	Wax printing	Distance based	*Adulteration—potassium iodate in salt/Milk* ^165^
	Wax rubbing	Colorimetric assays	*Adulteration—starch in milk* ^166^
	Screen printing	Colorimetric assays	*Quality control—food additives—nitrite and nitrate* ^167^
	Inkjet printing	SERS	*Quality control—food additives—colorants from the skin of dals and vegetables* ^168^
2020	Hybrid—cutting/silanization	Colorimetric assays	*Heavy metals—mercury in fish/water* ^169^
	Screen printing	Colorimetric assays	*Quality control—food additives—nitrite and nitrate* ^170^
	Wax printing	Colorimetric assays	*Quality control—food additives—borax, salicylic acid, nitrite and nitrate* ^171^
	Wax printing	Hybrid/colorimetric assays	*Adulteration—ketamine in beverages* ^172^
	Laser printing	Colorimetric assays	*Quality control—food additives—tartrazine and indigo carmine* ^173^
	Wax printing	Colorimetric assays	*Neurotoxic pesticides—phoxim, carbaryl, carbofuran, methamidophos, chlorpyrifos, triazophos* ^174^
	Wax printing	Colorimetric assays	*Antibiotic residue—Norfloxacin in meat* ^175^
	Wax printing	Colorimetric assays	*Quality control—food additives—nitrite in pork* ^176^
	Photolithography	Colorimetric assays	*Mycotoxin—aflatoxin B1 in milk* ^38^
	Wax printing	Chemiluminescence	*Drug Residue—beta-agonists in meat* ^177^
2021	Wax printing	Distance based	*Chemical residue—bromide and bromate in water* ^178^
	Screen printing	Colorimetric assays	*Heavy metals—Cr^3+^ water* ^179^
2022	Wax printing	Colorimetric assays	*Biotoxin—microcystin in water* ^180^
2023	Hybrid—cutting /lamination	Hybrid/colorimetric assays	*Antioxidant residue—gallic acid or oenotannin in fruits* ^181^
2024	Laser printing	Colorimetric assays	*Adulteration—syrups in natural honey* ^182^
	Wax printing	Colorimetric assays	*Pesticide residue—butachlor in mung beans* ^183^

## IV. μPAD FLUID FLOW CONTROL TECHNIQUES

In this section, our review is focused on the progress that has been made in the area of fluid flow control methods in μPADs and the different tools that are developed to integrate the methods into paper devices.

### A. Theory

For the development of accurate and predictable μPADs, understanding the flow behavior is important. Several studies have been reported incorporating the flow control functionality in μPADs. Fluid flow in a porous medium depends on a number of parameters including the pore size, the liquid-air surface tension, and the liquid–solid contact angle. The capillary action in paper-based devices can be formulated by Lucas–Washburn's equation,^184,185^

$$L\;=\;\sqrt{\frac{\gamma Dt\cos\theta }{4\mu }},$$ (1)

where *L* is the distance traveled by the fluid under capillary force in paper channel, *t* is the time, *D* is the average pore radius of the paper matrix, μ is the viscosity of the fluid, γ is the surface tension, and θ is the contact angle. This equation assumes one-dimensional flow in a homogenous porous membrane and cannot be used to accurately predict the flow rate in real devices.

Darcy's law can be used to describe the fluid flow in a paper fluidic network containing multiple channels and multiple porous substrates,^186^

$$Q=\;\frac{kA}{\mu L}\Delta P,$$ (2)

where *Q* is the volumetric flow rate, *k* is the material permeability, *A* is the cross-sectional area of the paper substrate, and *P* is the pressure difference over the medium length.

Effects of evaporation play an important role in liquid flow in paper substrates and, thus, leading to a new expression for flow length as developed by Liu *et al.*,^187^

$$h_{ev}=\;\frac{m_{e}}{\rho \varepsilon w\delta },$$ (3)

where $m_{e}$ is the predicted wicking liquid mass with evaporation, ρ is the density of the fluid, ε is the substrate's effective porosity, *w* is the channel width, and δ is the thickness of the substrate. Enclosed paper channel devices have a faster flow rate compared to open devices, due to the effects of evaporation.^188^ Depending on the applications, a flow rate control in paper devices is essential for many applications and several methods were developed that permitted a controlled flow. These methods can be categorized into three major ones, according to the operation modes, as explained below.

### B. Mechanical-based methods

Physical motion of components is used to achieve connection or disconnection to the channel surface thereby controlling the flow of the fluid. Research has been carried out on temperature-controlled valve system, in which the fluid flow is guided by melting wax in the patterned channel by heating.^189^ Kong *et al.*^190^ developed a reconfigurable actuator device made out of a folded chromatography paper, which was activated by fluid addition either at the crest or trough, engaging or breaking the fluidic contact between channels.

**FIG. 9.**
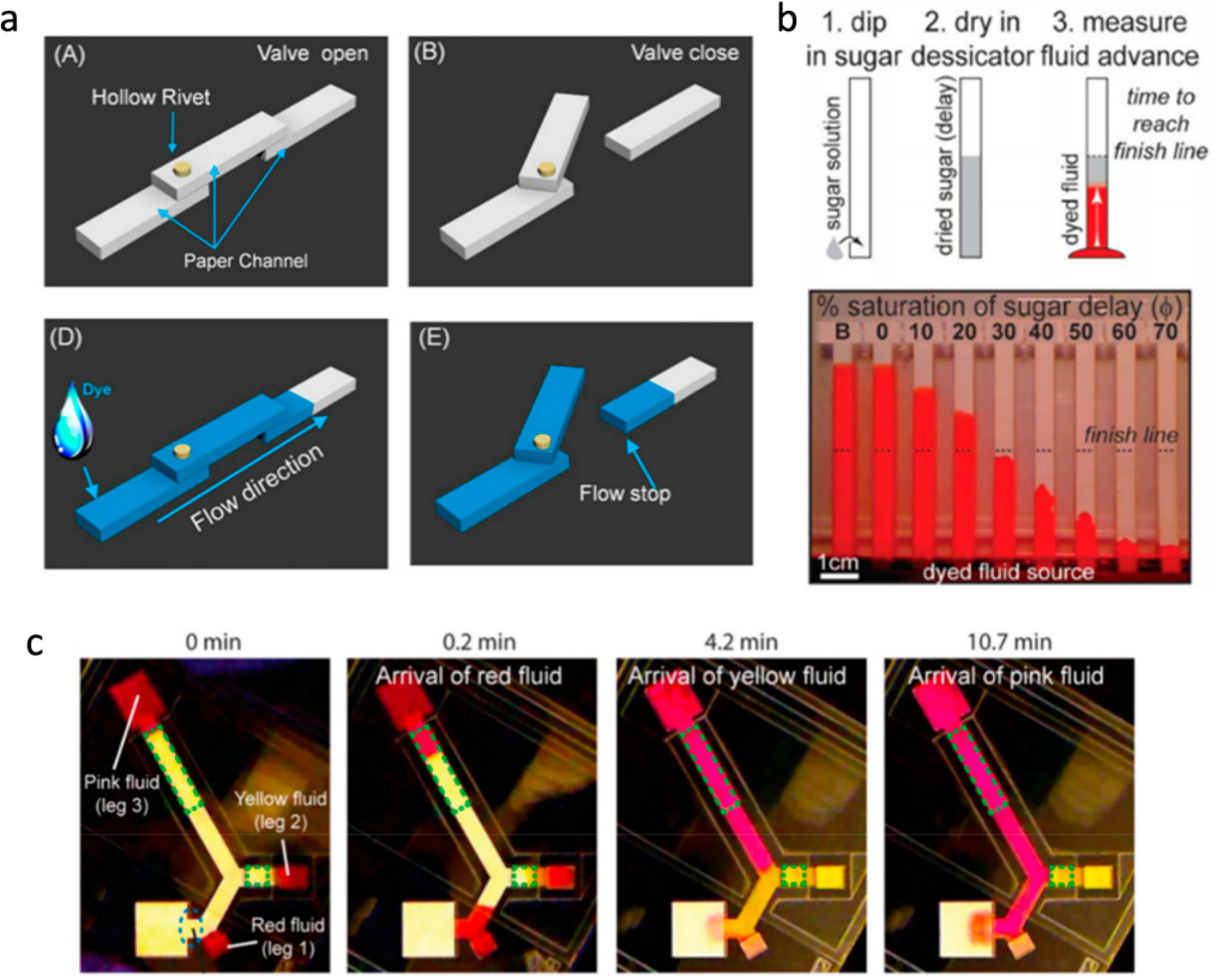
(a) Schematics of the hollow-rivet-assisted movable valve paper device, reprinted from Ref. 191. (b) Preparation steps of delayed strip using sugar solution—experimental images of the flow test with varying concentrations of sugar solution, the dashed line, and strip “B” indicate the finish line and an untreated strip, respectively, reprinted from Ref. 36. (c) Sequential delivery of three colored fluids using cellulose shunts, reprinted from Ref. 194.

Rotational valves on paper-based analytical devices have been implemented using hollow rivets,^191^ plastic comb binding spines,^192^ and rotational paper-based microfluidic chips^193^ as ways to control the connection or disconnection between the detecting zones and fluid channel, as shown in Fig. 9(a). Expandable material (sponge) has been demonstrated to control the fluid low as demonstrated by Toley *et al.*^195^ When the sponge gets wet with the fluid flow, it expands resulting in either stopping the flow or transferring the flow to another channel, in turn acting as a switch. An electromagnetic valve on μPADs, made by applying ferromagnetic nanoparticles, has been studied. Here, electromagnets are used to operate the connection and disconnection of the valves.^196^ Kong *et al.*^190^ proposed an actuator device made out of a folded chromatography paper, which was actuated by fluid addition either at the crest or trough, engaging or breaking the fluidic contact between the channels.

### C. Chemical-based methods

The wicking properties of the paper can be varied by simply embedding various chemicals in the paper channel. A fluid delay effect is generated by injecting dissoluble solutions like sucrose^36^ in the paper strip and letting it dry, resulting in a viscosity change [see Fig. 9(b)]. Programmable flow delays can also be generated by dissolvable bridges, making use of the dissolving property of pullulan films.^197^ An increased flow rate is demonstrated by printing toner with hydrophobic properties on the top and bottom of the paper device, thereby preventing the fluid evaporation.^198^

Coating hydrophobic paraffin wax on fluid channels can control fluid flow by stacking each micropatterned layer and interposing paraffin wax-patterned layers between them.^199^ Studies on fluid manipulation technology using surfactants have also been developed. Chen *et al.*^200^ developed a fluid diode technology in which dried surfactants have been used to bridge a hydrophobic gap in the flow path. However, using dissolving materials in paper channels may affect samples or downstream reactions. Strong *et al.*^201^ attempted to overcome this limitation by introducing wax printed fluid time delays on the top and bottom of pre-fabricated μPAD channels.

### D. Geometry-based methods

In this method, flow rate control is achieved by changing the channel length, width, or flow path. A lot of research has been done on controlling the flow rate by changing the width and length of the paper channel. Fu *et al.*^202^ showed that the flow rate decreases as the width of the paper becomes wider. This is because the longer the width and length of the paper, the greater the resistance and the slower the fluid velocity. The baffle design by Apilux *et al.*^203^ increased the flow length thereby creating a time delay resulting in the sequential reagent flow to the detection region. Fu *et al.*^202^ placed different reagents at different distances from the detection zone, thereby generating sequential multiple flows to the detection zone.

Applying pressure on specific regions of the paper is another reported way of controlling the fluid flow. The pressure adjusts the flow rate by reducing the pore size and cross-sectional area on the paper, thereby increasing the fluid resistance creating a fluid delay.^204^ In another method, the use of an absorption pad generates the delay in the fluid flow by placing it in positions where the fluid is to be delayed. It has been shown the delay time can be varied by controlling the dimensions of the pad,^194^ as shown in Fig. 9(c). The sandwiching paper channel between two flexible films prevents sample evaporation thereby accelerating the flow rate.^188^ The advantage of this method is that the increased velocity of the fluid can reduce the diagnostic time.

## V. DETECTION METHODS USING μPADS

**FIG. 10.**
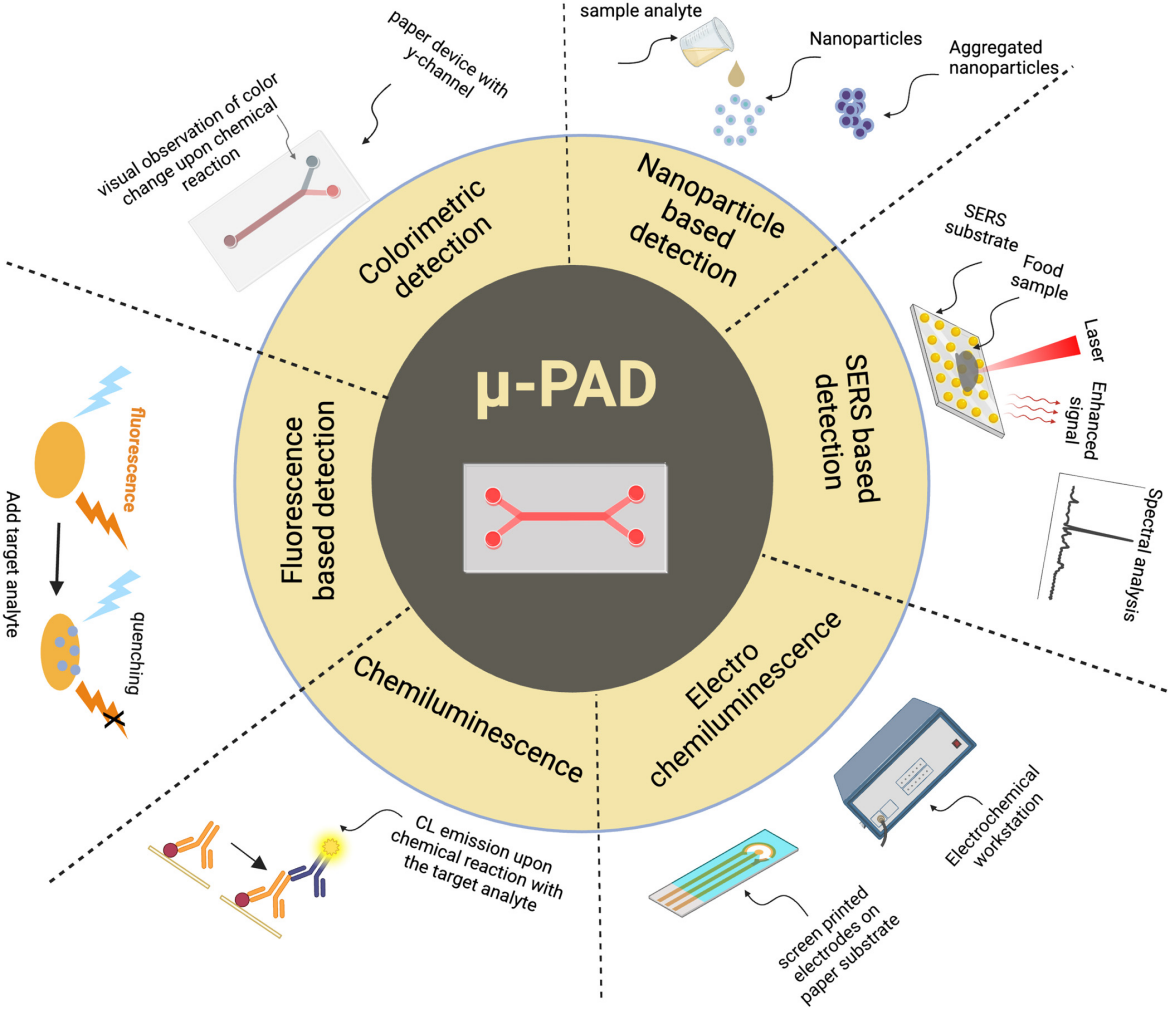
Different detection methods using paper-based microfluidic devices for food safety analysis.

Food safety analysis is one of the major applications of μPADs that requires the development of low cost, rapid, and portable detection methods for rapid monitoring incessant. This section reviews the recently reported μPADS that employed different detection methods, such as colorimetric, electrochemical, chemiluminescence, fluorescence, nanoparticle, and SERS (surface-enhanced Raman scattering) based detection techniques for food and water analysis (Fig. 10).

### A. Colorimetric detection

Colorimetric detection is the most widely used instrument-free detection method for μPADs.^205^ It relies on the observation of color change during a chemical reaction between the desired analyte and an indicator agent, where the results can be qualitatively analyzed by visual monitoring or quantitatively by using tools like handheld scanners, cell phone,^206^ or digital cameras^207^ to measure the intensity of the color produced. Morbioli *et al.*^208^ addressed the different methods to improve the color generation using nanoparticles, dyes, enzymes, etc.

A proof of concept study for a simple paper-based test was demonstrated for the specific detection of *Escherichia coli* (*E. coli*) bacteria in environmental samples using colorimetric detection.^209^ A patterned paper chip was used for the realization of a visible immune assay by immobilizing the antibodies for capturing *E. coli* in the detection zones of the paper chip.^94,138,210^ Numerous studies have reported the use of μPADs associated with colorimetric detection for the determination of food additives like nitrite and nitrate in food samples.^54,137,211^ Cardoso *et al.*^51^ performed the colorimetric detection of nitrite through the modified Griess reaction.^212^ Here, the μPAD devices were fabricated by stamping process in a geometry containing eight circular detection zones with immobilized reagents to produce selective color changes when the sample is placed and one central zone as sample inlet. The presence of nitrite in meat samples was detected using μPADs imprinted with wax material, reaction between Griess reagent in the test zone and nitrite in meat samples produced colored complexes.^53^

μPADs based on colorimetric detection have been demonstrated for the detection of the presence of pesticide residues in food products.^213^ Nouanthavong *et al.* reported the use of μPAD with nanoceria for the detection of organophosphate (OP) pesticides using an enzyme inhibition assay with acetylcholinesterase (AChE) and choline oxidase (ChOX).^146^ A significant interest was developed in analyzing milk and milk products for quality testing. A microfluidic paper-based ELISA platform was used to detect clenbuterol, an illicitly used feed additive for animals,^67^ by measuring the intensity of color change that was proportional to the analyte concentration. Au nanoparticle (NP) coated paper substrates were used for the detection of melamine in milk samples, the presence of which can cause a variety of health risk issues to infants. The interaction between melamine and the NPs produced a wine red to blue color change, measured using UV-VIS spectroscopy.^55^ A study was conducted by Salve *et al.* for the detection of urea, starch, salt, and detergent in milk samples using μPADs fabricated with polydimethylsiloxane (PDMS) as a hydrophobic barrier to confine reagents specific to the target analyte.^214^

Several works were reported toward the detection of heavy metals in water, using a colorimetric paper sensor. A novel approach for rapid and sensitive detection of heavy metals using μPAD was demonstrated by Hossain and Brennan with solgel entrapped reagents to allow colorimetric visualization of the enzymatic activity of beta-galactosidase (B-GAL).^134^ A smart gold nanosensor based μPAD was designed to detect very low concentrations of Cu^2+^ and Pb^2+^. A visible blue color was developed due to the formation of NP aggregates upon binding with metal ions.^215^ A novel, highly sensitive and selective μPAD for the detection of Cu^2+^ in groundwater, drinking water, rice, etc. was achieved by Chaiyo *et al.*^145^ The presence of Hg (II) in tap and commercial bottled water was detected by a paper-based device using silver nanoplates, the color of the nanosilver in the test area changes in the presence of Hg(II).^216^

In spite of all these demonstrated applications, achieving accurate detection with the naked eye is challenging due to the inhomogeneity of the color distribution on the paper assay.^217^ High background noise of the paper or the sample is another disadvantage of colorimetric detection resulting in low detection limits.

### B. Electrochemical detection

Electrochemical μPADs (ePADs) using electrochemical detection is another commonly used method for the detection of food adulterants that involves the direct conversion of a biological or chemical signal to an electrical one to enhance the analytical performance of μPADs. This method comprises a three-electrode system: a counter, working, and reference electrode, which are deposited in the form of conductive inks (silver or carbon inks) on the paper matrix.^218^ Samples are added to the hydrophilic region of the paper surrounded by the hydrophobic barrier. The wicking property of the paper is used to flow the sample to the sensing zones with electrodes (modified with some reagents) and a precise signal is obtained from the redox reaction involving the sample and the reagents.

A low-cost green biosensor consisting of a hydrophilic paper disk with immobilized glucose oxidase, placed in screen-printed carbon electrode, was used for glucose concentration determination.^47^ Microwire electrodes, as an alternative to screen-printed electrodes, are employed in ePADs for the non-enzymatic detection of glucose, fructose, and sucrose in aqueous solutions.^219^ Direct-writing of electrode on paper was demonstrated by Li *et al.*,^220^ using a pressure-based ball pen to form ePAD to detect melamine in food samples. Pencil drawn electrodes were employed for the detection of analgesics and sedation drugs in whiskey samples, using ePADs.^161^ Spiking whiskey with these drugs has been a common practice to prevent hangover and unconsciousness. The integration of μPADs with zeolite-nanoflakes and graphene oxide nanocrystals was reported to be used for the electrochemical sensing of ketamine in alcoholic and non-alcoholic drinks.^221^

Most electrochemical detections are still performed in the lab due to the need for heavy equipment. Even though handheld-potentiostat equipment are available, they need to be miniaturized and integrated into lab-on-a-chip platforms for use in limited resource setting areas.^222^

### C. Chemiluminescence detection

The chemiluminescence (CL) mechanism is based on the emission of light as a result of a chemical reaction. The luminescence in this method is controlled by the mixing of fluid flow reagents. CL based detection method uses inexpensive reagents and is characterized by a high signal to noise ratio and low limits of detection.^223^ However, the measurement using this method needs to be done in the dark making the detection process complicated.^224^

A common CL system being employed in μPADs is the luminol-H_2_O_2_ reaction system. In this system, transition metals, enzymes, and NPs have a good catalytic effect making it useful for the detection of various target analytes.^225^ Li *et al.*^226^ developed a double layered 3D μPAD for the detection of glucose, lactate, cholesterol, and choline, based on the appearance of temporally resolved peaks corresponding to the reactions between luminol and H_2_O_2_ upon the addition of different analytes. Alahmad *et al.*^147^ developed a device to detect Cr(III) in natural water samples based on luminol oxidation by H_2_O_2_ in the presence of Cr(III). Wang *et al.*^227^ fabricated paper-based molecular imprinted polymer (MIP) grafted multi-disk micro-disk plate that can provide the possibility of performing analytical assays, using MIP as a recognition element and enzyme catalyzed CL as the detection method to determine the presence of pesticides in liquid food samples. A paper-based chemiluminescence device was fabricated by Liu *et al.*^142^ for quantifying the level of dichlorvos (a type of insecticide) in vegetables without complicated sample pretreatment.^142^ Chemiluminescence assay has also been employed for the determination of food quality. For example, Hassanzadeh *et al.* developed a paper-based chemiluminescence device for the estimation of total phenolic antioxidant capacity in molasses and honey samples.^228^

Even though CL based detection method has great potential in food safety analysis, the intensive sample pre-treatments, and the need for selective CL system specific to both analytes and samples, etc. are not currently suitable for paper-based ASSURED testing.^225^

### D. Fluorescence detection

μPADs based on fluorometric detection exploit the interaction between the target molecules and fluorescent dyes and measures the emission intensity during analysis under an excitation illumination. This technique has some advantages over other detection methods including low limit of detection, high specificity and sensitivity, and rapid and multiple detection of analytes in a cost-effective manner.^225^

Numerous fluorescent-based μPAD methods were reported for the detection of trace level amounts of heavy metal ions.^229–231^ An apparent fluorescent quenching of the test paper was observed when exposed to Hg^2+^, while a significant fluorescence enhancement was observed in the presence of Pb^2+^. Qi *et al.*^232^ used an ion imprinting technique to fabricate a 3D paper-based microfluidic device for the multiplexed detection of Cu^2+^ and Hg^2+^ ions based on the fluorescence quenching of CdTe quantum dots (QDs) and the results revealed good and reproducible performances for the analysis of real samples in local lake water and seawater. Fluorescence-based technique is a suitable method for the detection of allergens in food samples, as demonstrated by Weng and Neethirajan.^233^ A hybrid PDMS/μPAD aptasensor provided a rapid cost-effective and accurate determination of food allergens (egg white lysozyme, β-conglutin lupine, and brevetoxins in egg, sausage, and mussel, respectively). They used a specific aptamer-graphene oxide sensor that was coupled to QDs as the fluorescence label. Molecular-based fluorescence assay was employed by Ali *et al.*^234^ for the detection of *E. coli* in food products by employing a novel approach for isolating selective RNA cleaving fluoregenic DNAzymes (RFDs) on paper. A polydimethylsiloxane (PDMS)/paper/glass hybrid microfluidic system integrated with aptamer-functionalized graphene oxide was developed for multiplexed pathogen detection.^235^

The need for additional instrumentation, the significant background noise from the additives (to improve the whiteness in commercial paper), extensive sample preparation steps by trained personnel, etc. make the fluorescence detection method less suitable for paper-based devices that aim to be facile and low cost.^236^ Even though fluorescence sensing provides high specificity and selectivity, further improvement in the technique can be done with the cost-effective size reduction of fluorescence readers.

### E. Nanoparticle-based detection

Another sensing method for the detection of μPADs makes use of nanoparticles (NPs). The optical properties of NPs, due to their high plasmonic and catalytic efficiency,^237^ enable naked eye observation of any bio-recognition event. Many studies have proved that NPs are effective in enhancing the analytical performance of paper-based device.

Kudo *et al.*^238^ demonstrated the colorimetric detection of metal ions such as Zn^2+^ in environmental water, using a paper-based analytical device with water soluble cationic polymer (PDDA) NPs coated on the detection zone. The detection limit was found to be 0.53 *μ*M and, thus, represented a significant improvement over that achieved using commercial colorimetric Zn^2+^ test paper (9.7 *μ*M).^238^ There have been numerous other works^239–241^ that employed paper-based devices with NPs to enhance the detection efficiency for ion analysis. Gold NPs were used to functionalize μPADs for the detection of melamine in milk by observing a color change upon the addition of samples with contaminant residues.^55^ Kasoju *et al.*^38^ used an aptamer based colorimetric assay followed by salt induced aggregation of NPs for the detection of aflatoxin B1 in milk and animal feed. This device was shown to be an efficient tool for on-site detection of food toxins in less than a minute. Figueredo *et al.*^242^ used different types of nanomaterials, such as Fe_3_O_4_ NPs, multiwalled carbon nanotubes (MWCNT), and graphene oxide, to enhance the analytical performance of μPADs, thus solving the drawback of using additives avoiding possible interaction with possible enzyme activity. The modified μPADs allowed the visual detection of glucose at low concentrations.^242^ Kumar *et al.*^243^ and Núnez-Bajo *et al.*^244^ have also employed NPs for enhancing the detection performance of paper-based devices in glucose analysis.

Even though NP-based detection methods result in rapid detection even at low concentrations of analytes compared to the colorimetric method, it has some limitations. NPs can aggregate in colloidal solutions creating false negative results. The affinity of NPs for many proteins can cause false labeling. Also, this technique requires extensive sample preparations and needs a large number of solvents and reagents. These drawbacks make the NP-based detection method not very suitable for food safety applications using paper devices.^245^

### F. SERS based detection

Raman spectroscopy is an analytical technique based on inelastic light scattering and has many uses in determining the chemical and structural properties of different molecules. SERS enhances the Raman scattering signal of the analytes that are near or adsorbed on the surface of metal nanoparticles and offers high sensitivity, portability, and rapid detection up to a single molecular level.^246,247^ It is important to develop simple, flexible, and cost-effective substrates for broadening the SERS application window. Integrating SERS to mechanically stable and flexible paper substrates can provide a continuous flow condition for reproducible SERS measurements showing great potential for on-site analysis of food contaminants.^73^

One of the early studies based on this approach developed a paper-based device with Ag NPs and functionalized with negatively charged poly (sodium 4-styrene sulfonate) or PSS for the detection of common food dyes, such as sunset yellow and lemon yellow in drinks.^248^ Another research group used robust nanostructured-silver as flexible, paper-based SERS swabs for the direct detection of Metanil Yellow (MY) from toor dal (yellow split pigeon peas) samples and Malachite Green (MG) from green peas and green chilies. MY was efficiently detected in “spiked” dal samples with characteristics Raman peaks at 1148 and 1404 cm^−1^ with a detection limit of 1 *μ*M. Green peas and green chilies were also used to detect MG that showed a prominent peak at 1370 cm^−1^.^168^ Paper-based SERS strategy was also used for the detection of drugs in beverages such as estazolam (EST), where in the latter prepared aqueous solutions showed distinct peaks at 687 and 1000 cm^−1^.^249^ In a similar attempt, Ma *et al.*^250^ quantified the concentrations of pesticide residues like thiram, thiabendazole, and methyl parathion on apples, oranges, and tomatoes' surfaces using high density Ag NPs/GO on cellulose paper as SERS substrate. Various pathogens in food products have also been quantified using paper-based SERS. For instance, Tian *et al.* fabricated a SERS swab by functionalizing nitrocellulose membrane using gold nanorods for the determination of *E. coli* spread in spinach leaves.^251^

Although there are numerous research works reported using paper-based SERS technology in food analysis, there are still some challenges associated with it. For example, Ag-based SERS substrates have a limited shelf life due to the degeneration of the activity of silver.^168^ The SERS method has significant difficulties in excluding interference and target the compound of interest when analyzing complex samples.^249^ Developing cost-effective, target-specific, long-lasting, and reliable substrates, using specific target capture agents and removing the interreference are some possible strategies to increase the sensitivity and selectivity of SERS method for food safety analysis.

## VI. CONCLUSIONS AND FUTURE DIRECTIONS

The screening of hazardous materials in food is a major concern in the food safety sector. If the food quality control is not followed stringently, these harmful materials may lead to severe illness and mortality. Conventional food analytical systems rely on tedious, time consuming procedures for food hazard detection, in order to obtain reliable results. Hence, they are not always suitable for on-site rapid surveillance. World Health Organization standards for point-of-need diagnostic assay criteria are stated as: it should be Affordable, Sensitive, Specific, User-friendly, Rapid and Robust, Equipment-free, and Deliverable to end-users (ASSURED). Based on these criteria, every new assay system will be evaluated critically and a five-star scoring system will be used to assess new devices to be used in food safety testing systems.^252^ The recent advances in μPAD-based diagnostic devices and microfluidic technology potentially offer quick and dependable ASSURED surveillance options in food safety practices.

Detecting hazardous substances and pathogens in food is quite different from detecting those substances in liquid clinical samples such as blood or urine. The following refinements will be required for adopting this technology with maximum efficiency in food safety applications. For example, the food sample could be a solid piece of vegetable containing hard plant layers or a piece of processed meat fibrous muscle steak with raw and denatured proteins, containing hydrophobic lipids, insoluble fat, meat juices, salts, and spices added during cooking, also rich in many chemicals produced in the Maillard reaction during cooking.^253,254^ This complex nature of the food matrix restricts the availability of the target analyte to interact with the detection reagents. Also, depending upon the nature of the analyte in the food matrix and the μPAD matrix, strong physical and chemical interactions may interfere with the availability of the analyte to the detecting agent. Therefore, a typical food sample preparation involves mixing the food with a solvent, an extraction step to free the analyte from the food matrix, followed by a clean-up step to remove the interfering substances, and finally a concentration step or dilution step to be added if required. So, developments of easy methods for conversion of complex food samples into μPAD compatible liquid form will be a requirement for optimum μPAD assay performance.

Another significant area for improvement is to develop more sensitive detection and analysis systems for μPADs. Current detection methods mostly depend on traditional techniques and instruments such as scanners and grayscale image analysis. However, the conversion of colored image to grayscale hinders the advantages of colorimetry while multiplexing and reduces the assay sensitivity. In multiplexing, grayscale analysis of two different colors may detect with similar intensity. All the other methods, such as spectrometry and fluorescence-based assays, require expensive reagents and equipment limiting their potential for mass commercialization. For the advancement of μ-PAD field, the development of systems free of complex detection equipment is necessary.

We cannot conclude this review without mentioning some of the commercially available μPADs for food safety analysis and related applications. Elabscience based in Texas, USA has developed a number of lateral flow assays for detecting fungal toxins, toxic chemicals, pesticides, and antibiotics residues in food.^255^ R-biopharm, located in Germany, developed lateral flow devices (LFDs) consisting of immunochromatographic rapid test strips for allergen detection. Currently, they have LFDs for detecting peanut, hazelnut, coconut, almond, and mustard allergens. Also, they have dipstick (EZ PANGASIUS™ Pangasius Species Rapid Kit) tests for species detection in Pangasius fish. Their RIDA®QUICK CIS is an immunochromatographic test developed for the detection cow's milk in milk or cheese of other species (sheep and goat). They have LFDs, and paper-based screening cards for mycotoxin analysis.^256^ Merck and Australasian medical and scientific Ltd. and develop lateral flow tests for immunological detection of pathogens, such as *Salmonella*, *Listeria, E. coli*, and *Campylobacter* in food and environmental samples.^257,258^ Paperdrop Diagnostics S.L. based in Spain develops rapid diagnostic tests based on paper microfluidics. They have developed an assay called, Resistgene™ that offers a testing solution to detect multiple antimicrobial resistance genes from blood samples, focusing on the enzyme carbapenemase, a critical antibacterial resistance mechanism.^259^ Another important development in μPAD technology is I-Corps: A Paper-based Microfluidic Viral Diagnostic Device project by United States Department of Agriculture (USDA). I-Corps is based on the concept of using spectrum-shifting ability of gold nanoparticles as detection molecules on a passive paper flow assay.^260^ I-Corp can be used in many industries ranging from public health, veterinary medicine, food processing, and water testing.

The developments in the research field and commercial front show that the microfluidic paper analytic device (μPAD) technology is promising in food safety applications, especially as a simple, low-cost point-of-use testing platform. With continuing advances in fabrication techniques that enable mass production at lower costs, development of assays with enhanced sensitivity and specificity, further improvements in detection techniques that reduce or eliminate complex equipment, and commensurate progress in ancillary techniques for preparing μPAD-ready samples, μPADs are well positioned for widespread adoption in food safety industry.

## Data Availability

The data that support the findings of this study are available from the corresponding author upon reasonable request.
